# Banking on cooperation: an evolutionary analysis of microfinance loan repayment behaviour

**DOI:** 10.1017/ehs.2020.64

**Published:** 2020-12-14

**Authors:** Stefan Gehrig, Alex Mesoudi, Shakti Lamba

**Affiliations:** Human Behaviour and Cultural Evolution Group, Centre for Ecology and Conservation, College of Life and Environmental Sciences, University of Exeter, Penryn Campus, Cornwall TR10 9FE, UK

**Keywords:** Assortment, evolution of cooperation, group lending, joint liability, kin selection, microcredit, partner choice, reciprocity, social capital, social dilemma

## Abstract

Microfinance is an economic development tool that provides loans to low-income borrowers to stimulate economic growth and reduce financial hardship. Lenders typically require joint liability, where multiple borrowers share the responsibility of repaying a group loan. We propose that this lending practice creates a cooperation dilemma similar to that faced by humans and other organisms in nature across many domains. This could offer a real-world test case for evolutionary theories of cooperation from the biological sciences. In turn, such theories could provide new insights into loan repayment behaviour. We first hypothesise how group loan repayment efficacy should be affected by mechanisms of assortment from the evolutionary literature on cooperation, i.e. common ancestry (kin selection), prior interaction (reciprocity), partner choice, similarity of tags, social learning, and ecology and demography. We then assess selected hypotheses by reviewing 41 studies from 32 countries on micro-borrowers’ loan repayment, evaluating which characteristics of borrowers are associated with credit repayment behaviour. Surprisingly, we find that kinship is mostly negatively associated with repayment efficacy, but prior interaction and partner choice are both more positively associated. Our work highlights the scope of evolutionary theory to provide systematic insight into how humans respond to novel economic institutions and interventions.

## Graphical abstract

**Figure:**
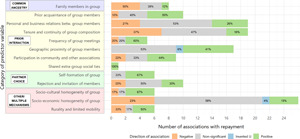

**Media summary:** The paper embeds an empirical review of group loan repayment in microfinance into an evolution-of-cooperation framework.

## 1. Introduction

Microfinance is a globally widespread economic development tool that aims to provide financial services to people from low-income backgrounds. One of its cornerstones is the provision of small, short-term loans which enable borrowers to invest in new income-generating activities (Armendáriz & Morduch, 2010; Brau & Woller, 2004; Cull & Morduch, 2018). Conventional lending by banks requires loan-takers to provide material collateral, such as a house or land, that the bank holds as a guarantee and will confiscate if the borrower fails to repay the initial loan. In contrast, microfinance institutions (MFIs) offer loans to individuals who cannot obtain loans from mainstream banking services because they cannot provide the required securities and have no formal credit rating.

A common feature of MFIs is to provide loans to a group of borrowers who are jointly liable to repay it. This has been called joint liability lending (JLL): borrowers are mutually responsible for each other's repayment and must collectively make up any shortfall in repayment if other group members are unable or unwilling to repay. Typically, if the group does not achieve repayment, all group members are barred access to future loans from the micro-lending programme. The typical economic rationale is that JLL can exploit local social networks and ‘peer pressure’ to boost repayment (Besley & Coate, 1995; Ghatak & Guinnane, 1999).

This interdependence of group members in JLL creates a cooperative dilemma, because the responsibility of collective loan repayment to the lender, as well as the potential benefit of loan renewal in case of success, is shared by all group members. Here we build on Lamba (2014) and propose that group-based lending provides a model system to test the extent to which evolutionary theories on cooperation from the biological sciences apply to contemporary human societies. Since JLL participants span the globe, this model system allows us to test evolutionary theories of cooperation in a diverse range of human populations within the same natural context. Conversely, evolutionary theories of cooperation from the biological sciences can potentially explain patterns of repayment observed in the microfinance sector.

In this paper, we (a) describe the cooperative dilemma presented by JLL (Section 2), (b) outline evolutionary mechanisms of cooperation and generate specific hypotheses about how these mechanisms may operate in the context of JLL (Section 3), (c) review empirical studies on microloan repayment to empirically assess whether their findings are qualitatively consistent with the evolutionary hypotheses that we derive for JLL (Section 4) and (d) discuss the results of the review and their implications for evolutionary biology and economics (Section 5). By connecting literatures from development economics and the evolutionary sciences that have to date developed separately, we identify a new model system, i.e. JLL, that can be used to test evolutionary theory on cooperation across a wide cross-section of humanity. Our study generates a range of questions, hypotheses and study designs that warrant further investigation by evolutionary biologists and economists.

## 2. Microfinance and the cooperative dilemma in joint liability lending

Microfinance describes a bundle of financial services of which the most common is microcredit, the aim of which is to provide loans to small-scale entrepreneurs to reduce financial poverty and spur economic growth. In recent decades, microfinance has grown and spread across the globe (Armendáriz & Morduch, 2010; Brau & Woller, 2004; Cull & Morduch, 2018). There are estimated to be over 200 million recipients of microfinance worldwide, three-quarters of whom are women, served by more than 3,000 MFIs, which include nongovernmental organisations, commercial banks and other financial institutions (Microcredit Summit Campaign, 2015). More than half of microfinance clients are living in ‘extreme poverty’ as defined by international poverty lines or the relative income position in their home country. Despite years of enthusiasm and success stories among practitioners and observers, rigorous evaluations of the impact of microcredit provision have at best shown modest causal effects on business and income growth (A. Banerjee et al., 2015; Duvendack et al., 2011; Meager, 2019) and debates about the evidence continue (Dahal & Fiala, 2020). Nevertheless, many scholars suggest that microfinance helps in ‘managing the ups and downs of lives in poverty and near-poverty, even when poverty persists’ (Cull & Morduch, 2018, p. 551) by improving household finances and smoothing consumption. This contributes to the well-being of borrowers, albeit not triggering transformative economic growth (although others point towards the potential harmful social consequences of microcredit, e.g. S. B. Banerjee & Jackson, 2017; Hulme, 2000).

The microfinance movement in its current form as an entrepreneur-targeted development intervention was initiated in the 1970s by the Grameen Bank in Bangladesh. One of the major innovations was the introduction of JLL in small groups, which is still central to many MFIs and often seen as key to their success (Armendáriz & Morduch, 2010; Hermes & Lensink, 2007). In the JLL model, individuals receive loans in groups and are liable for repayment as a collective: only when the group loan is fully repaid will the individual group members have access to future loans and avoid other penalties. This allows lending to customers who have little material collateral and lack formal records of creditworthiness. By allocating repayment responsibilities to a group rather than an individual, the JLL contract creates an incentive for group members to look after each other's financial investments and activities in order to maintain their own access to future loans. Consequently, information and enforcement duties of mainstream lenders are transferred to the borrowers themselves (Besley & Coate, 1995; Ghatak & Guinnane, 1999; Stiglitz, 1990), replacing material collateral with social collateral. As Armendáriz and Morduch (2010, p. 13) write, ‘the classic Grameen contract takes advantage of clients’ close ties within their communities’. However, this shared responsibility for repaying a collective loan creates a cooperative dilemma. Each borrower is better off not repaying their share of the loan and letting other group members repay it. Yet if all borrowers do not repay their share, then the group will fail to repay the collective loan and will face penalties including being blocked from future loans. Therefore, in order to repay the collective loan, it is essential for group members to cooperate, by paying in full their own share and by helping other group members to pay their share.

**Figure 1.**
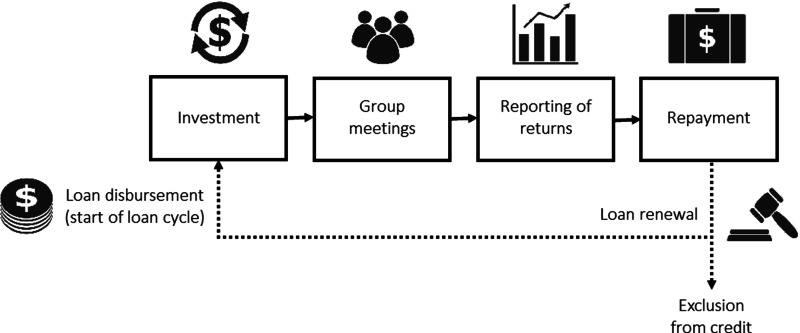
The stylised timeline of a typical joint liability loan cycle from loan disbursement by the microfinance institution (MFI) to the decision of the MFI about loan renewal or exclusion from credit, which is conditional on borrowers’ repayment behaviour. At each stage framed by a rectangle, borrowers can be thought of as taking an action and this action can affect the group's repayment outcome (see Table 1).

Figure 1 sketches the stylised timeline of a typical loan cycle and Table 1 describes the decisions taken by group members at each step. These decisions all feed into the cooperative dilemma underlying JLL as there is conflict between the group goal (group's repayment) and the individual goal (maximise own income). For example, the decision of a borrower regarding how to spend the loan, whether to monitor others’ investments, how many group meetings to attend or whether to truthfully report the outcomes of one's own project, can all affect whether the loan is fully repaid at the end of the loan cycle.

**Table 1.** Description of typical stages of a microfinance loan cycle with joint liability lending (see Figure 1) and how group members’ decisions at these stages affect the cooperative dilemma.

Stage	Description	Decision from borrower's perspective	How the decision affects the cooperative dilemma of JLL
Loan disbursement	Credit money is given to group members	—	—
Investment	Group members invest their share of the loan in projects of their choice	How to invest the loan and whether to monitor others’ investment	Temptation for short-term consumption of the loan money, poor project choice or low entrepreneurial effort can reduce the likelihood of successful group repayment, but can be in the immediate self-interest of the borrower. Group members can monitor each other's investments, but there is also an incentive to free-ride on the monitoring efforts of others
Group meetings	Group members participate in regular meetings to discuss loan-related issues and repay small instalments	Whether to attend group meetings and monitor other group members	Meetings confer multiple benefits to the loan group by providing opportunities for mutual monitoring, exchange of business-related information and training. Attendance can increase the likelihood of successful group repayment, but is individually costly (opportunity costs, travelling, being audited by lender, monitoring others)
Reporting of returns	Members disclose the returns from their projects	Whether to be honest about investments and returns	Investment outcomes are reported in order to determine the ability to repay. Reporting lower returns than realised can increase individual income, especially if it is costly for the lender or other group members to verify. This benefits the individual but decreases the likelihood of group repayment
Repayment	Group members contribute their monetary share to repay the group's loan	Whether and how much to contribute to loan repayment and whether to sanction other noncontributors	Not contributing can increase own income when others help out and repay one's missing share. Social sanctions may prevent this, but if sanctions are costly to impose, there is an incentive to free-ride on the sanctioning efforts of others
Loan renewal/Exclusion from credit	The group is either in default and excluded from future loans by the lender, or repays and becomes eligible to enter another loan cycle, often with improved conditions like larger loans	—	—

Despite the cooperative dilemma at the heart of JLL, repayment rates of Grameen Bank and other MFIs employing the JLL model have been high, often above 95% (Armendáriz & Morduch, 2010; Brau & Woller, 2004; Cull & Morduch, 2018; Hossain, 1988). Yet there is considerable variation in repayment rates across time and space. For example, JLL credit defaults temporarily rose from around 2% to 90% in the 2010 Indian microfinance crisis (Haldar & Stiglitz, 2016). There is also variation in the reported success of the JLL model relative to individual liability micro-lending. In some contexts, empirical studies report that JLL leads to better loan repayment than when individuals are solely liable for the loan (Carpena et al., 2013; Gomez & Santor, 2008; Khandker, 2012; Mahmud, 2019), while in others there is no difference (Attanasio et al., 2015; Giné & Karlan, 2014). This variation in repayment rates and the variable success of the JLL model is difficult to explain.

We suggest that variation in repayment rates and therefore the success of JLL may be linked to whether individuals can solve the cooperative dilemma underlying it. If this is the case, then we expect that factors facilitating cooperation amongst group members should have a positive outcome on repayment efficacy. Here we propose and test whether these factors may be the same as those proposed to facilitate the evolution of cooperation in nature as identified in the biological sciences.

The notion that repayment under JLL is a public good, requiring cooperation and collective action, has been put forward by some social scientists (Anthony, 2005; Guttman, 2007; Sabin & Reed-Tsochas, 2018; van Bastelaer & Leathers, 2006), but it is not always treated explicitly as such in group-lending models. For example, the seminal economic model of group lending by Besley and Coate (1995) assumes that borrowers employ sanctions to oblige other group members to repay their shares, making repayment the self-interested choice. However, while sanctions are assumed to be caused by the ‘social connectedness’ of group members, how this social connectedness arises is not addressed. Similarly, peer punishment and ‘social capital’ – the ability to trust and cooperate with peers owing to some social structure, relations or norms (Coleman, 1988) – are widely cited reasons for the success of group lending (e.g. Hermes & Lensink, 2007), but how and why certain social structures help to solve the cooperative dilemma inherent to JLL is unspecified. We propose that evolutionary theories of cooperation provide a tractable and measurable framework for understanding concepts like social capital and social connectedness, and may help to unify the currently diverse and disconnected findings in the literature on JLL.

## 3. Evolutionary theories of cooperation and predictions for microfinance loan repayment

### 3.1. The puzzle of cooperation and a general solution

In evolutionary biology, cooperation is defined as a behaviour that is beneficial to the recipient of the act, often (but not necessarily) at a cost to the actor (West et al., 2007a). The existence of costly cooperation presents a puzzle to evolutionary biologists. The problem arises because cooperators interact with selfish individuals (i.e. ‘defectors’) who benefit from the acts of cooperators but never bear the costs of cooperating themselves. Over the long term, selfish individuals should outcompete cooperators because selfish individuals accrue benefits while cooperators accumulate costs.

A general solution to this puzzle is to ensure that cooperators preferentially interact with other cooperators in the population (Fletcher & Doebeli, 2009; Fletcher & Zwick, 2006; Queller, 1985). This allows cooperators to share and accumulate the benefits of cooperation amongst themselves and prevents selfish individuals from free-riding on these benefits. Hence, the evolution of cooperation requires mechanisms of (positive) assortment that ensure that the average interaction group of cooperators contains more cooperators than the average interaction group of defectors (Fletcher & Doebeli, 2009). Assortment prevents defectors from gaining the benefits of cooperation.

The following frequently used mathematical formalisation popularly known as ‘Hamilton's rule’ (Hamilton, 1964a) specifies the conditions under which cooperative behaviour is favoured:
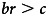


Cooperative behaviour is favoured when the benefit *b* of cooperation to the recipient, scaled by the degree of ‘relatedness’ *r* between the actor and the recipient, is greater than the cost *c* of cooperation to the actor. In Hamilton's original formulation (Hamilton, 1964a), *r* was interpreted as the degree of genetic similarity between the actor and the recipient. However, *r* can be more broadly interpreted as the degree to which cooperators in the population interact preferentially with other cooperators (Fletcher & Doebeli, 2009, 2010; Fletcher & Zwick, 2006; Queller, 1992), as it is the statistical association between like types (Hamilton, 1975). Hence, *r* is a measure of assortment.

### 3.2. Assortment mechanisms and evolutionary hypotheses for loan repayment

Over the last half-century, several mechanisms of assortment have been proposed that support the evolution of cooperation (for reviews, see Lehmann & Keller, 2006; Nowak, 2006; Sachs et al., 2004; West et al., 2007b) and an extensive literature has explored their relevance to humans (e.g. Apicella & Silk, 2019; Hammerstein, 2003; N. Henrich & Henrich, 2007; Powers et al., 2019; Rand & Nowak, 2013). Here, we describe a subset that we consider important in the context of microfinance. For each assortment mechanism, we derive hypotheses for loan repayment, some of which we then test in Section 4. As argued in Section 2, the basis for our predictions is the assumption that higher repayment efficacy in JLL groups, i.e. lower likelihood of credit default or loan delinquency, constitutes a higher degree of cooperation among group members.

#### 3.2.1. Common ancestry

Cooperation can be maintained when it is preferentially directed towards genetic relatives of the actor (Hamilton, 1964a, b). Here, the mechanism of assortment is genetic. The likelihood of two individuals sharing a gene that makes them behave cooperatively increases with increasing genetic relatedness (Grafen, 2007, 2009). For example, a gene for cooperation is more likely to be shared by identical twins than fraternal twins. Thus, common ancestry is a reliable indicator that the recipient of cooperation is also likely to be cooperative and pass on the benefits of received cooperation to future generations of kin. This process by which cooperation evolves owing to the actor and the recipient sharing ancestry is called ‘kin selection’ (Maynard Smith, 1964). Interacting preferentially with kin is a heuristic that allows the assortment of cooperators and can lead to an accumulation of the benefits of cooperation across generations. The strength of kin-directed cooperation is therefore affected by the degree of genetic relatedness between the actor and recipient. For example, siblings, who on average share 50% of unique genetic variation, should be more likely to help one another than cousins, who share 12.5%.

Kin selection requires the availability of information about common ancestry which can be gained from cues and psychological short-cuts that identify kin (Bressan & Kramer, 2015; Lacy & Sherman, 1983). It leads to the evolution of emotions and motivations to care for kin, which are observed in human populations across the world (Ko et al., 2019). There is ample evidence that people bias help towards close kin for many different types of interaction such as food sharing (Gurven et al., 2002; Kaplan et al., 1985), cooperative hunting (Morgan, 1979), monetary giving (Bowles & Posel, 2005) and child care (Anderson et al., 1999).

##### Hypothesis for microfinance research

JLL groups comprising individuals who are more closely related via common ancestry (i.e. genetic kin) should have higher repayment efficacy than those comprising non-kin.

#### 3.2.2. Prior interaction

Cooperation can be maintained when the actor's decision to cooperate or defect depends on the previous cooperative behaviours of the recipient. This is called reciprocity and can be based either on prior interactions with the same partner (direct reciprocity; Axelrod, 1984; Axelrod & Hamilton, 1981; Trivers, 1971) or on the basis of one's partner's reputation from her prior interactions with other partners (indirect reciprocity; Alexander, 1987; Leimar & Hammerstein, 2001; Nowak & Sigmund, 2005; Panchanathan & Boyd, 2003). Assortment arises because individuals direct cooperation to others who are known to have cooperated in the past. In this case, assortment increases with the number of repeated interactions or with more reliable information on the behaviour of others (Nowak, 2006). The idea that repeated interactions can facilitate outcomes (e.g. cooperation) which do not arise in one-shot encounters is well known to economists as the folk theorem (Fudenberg & Maskin, 1986). Direct reciprocity requires cognitive mechanisms such as memory to identify suitable long-term partners. It leads to the evolution of emotional motivations such as trust, affinity and sympathy to maintain reciprocal relationships (Fessler & Haley, 2003; Seyfarth & Cheney, 2012). Indirect reciprocity in humans is probably supported by our capacity for language to pass on information (Nowak & Sigmund, 2005), inclinations to spread gossip (N. Henrich & Henrich, 2007), concern with reputation (Karlan and McConnell, 2014; Milinski, 2016) and sympathy for people who do good to others (Bereczkei et al., 2007). There is evidence from laboratory experiments and field studies that humans reciprocate directly (Dal Bó & Fréchette, 2018; Fischbacher et al., 2001; Gurven, 2006; Kasper & Borgerhoff Mulder, 2015) and indirectly (Diekmann et al., 2014; Seinen & Schram, 2006; Wedekind & Braithwaite, 2002).

##### Hypotheses for microfinance research

JLL groups with the opportunity for direct reciprocity between group members, such as when the group participates in repeated loan cycles (Che, 2002), should have higher repayment efficacy. Opportunities for mutual exchange outside the JLL group, such as when members have personal and business relations with each other (Anthony, 2005; de Quidt et al., 2016), should be associated with higher repayment efficacy. JLL groups with the opportunity for indirect reciprocity, such as when members share mutual acquaintances within a larger social network (Postelnicu et al., 2019), or engage in social activities outside the group, should have higher repayment efficacy as this may provide a means to acquire and spread reputational information.

#### 3.2.3. Partner choice

Cooperation can be maintained when actors are able to select their interaction partners. Assortment occurs because individuals can choose to interact with known cooperators and avoid interacting with defectors from the outset (Bull & Rice, 1991; Enquist & Leimar, 1993; Gintis et al., 2001; Hammerstein & Noë, 2016; Roberts, 1998). This is different to, but can work alongside, other mechanisms such as reciprocity (Section 3.2.2), in which individuals do not actively choose their interaction partners or ‘walk away’ from them, but can only choose whether or not to cooperate with an encountered partner (partner choice vs. partner control, see Schino & Aureli, 2017). Partner choice requires the ability to identify and choose cooperative partners using signals of their likelihood of being a cooperator. Signals of likely cooperation should be costly, and thereby ‘honest’, so that defectors cannot fool cooperators by faking such signals (Bowles & Hammerstein, 2003). Honest signals might include an individual's past cooperative behaviour (Guido et al., 2019; Sylwester & Roberts, 2010), costly cultural practices such as holding extravagant feasts (Barker et al., 2019), or facial or other physical characteristics (although evidence on humans’ capacity to detect cheaters by biological markers alone is mixed; Aamodt & Custer, 2006; Fetchenhauer et al., 2010).

##### Hypotheses for microfinance research

JLL groups which form via the selection of group members by other members, e.g. by being able to choose who is admitted to your group, should have higher repayment efficacy than groups where membership is not controlled by group members. Note that self-formation of groups is a traditional feature of the Grameen Bank, with economists suggesting that this allows peers to screen each other for the riskiness of their business project (Armendáriz & Morduch, 2010; Ghatak & Guinnane, 1999; Van Tassel, 1999). We expand this idea by suggesting that borrowers might screen for cooperativeness in general (as in biological market theory, see Hammerstein & Noë, 2016).

#### 3.2.4. Similarity of tags

Cooperation can be maintained when it is directed only towards interaction partners that are similar to the actor in an arbitrary observable character or tag if that tag is a reliable indicator that the recipient of cooperation is also a cooperator (Antal et al., 2009; Gardner & West, 2010; Hales, 2005; Hammond & Axelrod, 2006; Riolo et al., 2001). In this case, interacting based on this tag leads to assortment. Tag-based cooperators of this type have sometimes been referred to as ‘greenbeards’, after a thought experiment in which green beards act as tags of cooperation (Dawkins, 1976; Gardner & West, 2010). Assortment via tags is threatened when defectors can fake the cooperators’ tags and thus requires, and increases with, the reliability of tags as indicators of behaviour (Hales, 2005), for example by using signals that noncooperators cannot easily fake. Tag-based cooperation is different to partner choice (Section 3.2.3) as individuals cannot actively choose their interaction partners, but can only choose whether or not to cooperate with an encountered interaction partner. In humans, tag-based cooperation depends on a psychological inclination to cooperate preferentially with others who appear similar in certain ways (Fu et al., 2012). Such tags might include accents (Cohen & Haun, 2013), facial resemblance (DeBruine, 2002), expressions of cultural and social identity like clothing, humour, sports or music preferences (Smaldino, 2017) or other arbitrary markers of groups (Tajfel & Turner, 1986).

##### Hypotheses for microfinance research

Repayment efficacy may be structured by observable biological or socio-cultural tags (e.g. accents, facial resemblance, clothing), after controlling for other differences between groups. Assuming that the tag is a known and reliable indicator of cooperation, a more specific prediction is that homogenous JLL groups whose members share this tag should have higher repayment efficacy than heterogenous groups whose members have different tags, after controlling for other differences between groups.

#### 3.2.5. Social learning

Cooperation can be maintained when cooperative behaviour is transmitted between individuals via social learning mechanisms such as imitation and conformity (Boyd & Richerson, 1985; J. Henrich & Boyd, 2001; N. Henrich & Henrich, 2007). Socially learned rules or norms of behaviour can make individuals in an interacting group more similar to each other (Apicella & Silk, 2019; Boyd & Richerson, 2009). Hence, the assortment of cooperators can be achieved via social learning. Tendencies to conform to others’ behaviour and adopt local institutions can generate some groups that are more cooperative and others that are less so (Chudek & Henrich, 2011). Then, if there is competition between these groups and a more cooperative group stands to outcompete a less cooperative group, cooperation can emerge as the costs borne by the individuals of a more cooperative group are outweighed by the benefits they receive when their group wins and survives. This process has been called cultural group selection (Boyd & Richerson, 1985; J. Henrich, 2004; Richerson et al., 2016; Waring et al., 2015) and the degree of assortment increases with the tendency of individuals to socially learn and exhibit the behavioural rules of the group they are in, compared with other groups. Note that any type of assortment mechanism, including the ones mentioned in previous sections, can be conceptualised as resulting in group selection because assortment leads to ‘groups’ (e.g. kin groups, groups of reciprocators) in which individuals preferentially bestow benefits on each other (see Birch, 2019; Wilson & Dugatkin, 1997; Wilson & Sober, 1994). Cultural group selection depends on socially learned norms sustained by conformity or punishment. While there is evidence of variation in cooperation across human populations (Gächter et al., 2010; J. Henrich et al., 2005; Richerson et al., 2016), which is consistent with assortment via social learning of cooperation, the degree to which such variation is caused by the social learning of behavioural norms rather than other factors, such as en masse individual responses to shared ecological and demographic factors which can also create behavioural uniformity, is debated (Lamba & Mace, 2011).

##### Hypotheses for microfinance research

Repayment efficacy may be structured by socio-cultural group if there are socially learned rules of cooperation associated with these socio-cultural group boundaries. Repayment efficacy may be structured at any level at which behaviour may be socially transmitted, i.e. community, village, ethno-linguistic group, any other kind of interaction group. Specifically, JLL groups of the same socio-cultural group should have more similar repayment rates to groups from a different socio-cultural group, after controlling for other differences.

#### 3.2.6. Ecology and demography

Cooperation can be maintained when ecological factors and demographic processes create and maintain behavioural similarity between individuals (Smaldino et al., 2013; Wilson, 1977). Assortment owing to ecology may arise because individuals’ environments vary with respect to the costs and benefits associated with cooperation. For example, the challenge of long-term survival in harsh ecologies may favour cooperation compared with groups living in less harsh ecologies (Smaldino et al., 2013) and moderate levels of ecological disturbance can aid cooperation relative to high or low levels of disturbance (Foster & Xavier, 2007). A substantial body of theory in evolutionary biology predicts that demographic characteristics of populations, such as their size and patterns of migration, are important drivers of cooperation and competition (Doebeli et al., 1997; Johnstone & Cant, 2008; Platt & Bever, 2009; West et al., 2002). Demography affects the stability of the various mechanisms of assortment described above, as well as the costs and benefits of cooperation. For example, high rates of migration in a population can destabilise cooperation by breaking down the assortment generated by common ancestry, prior interaction or partner choice. Similarly, cooperation via prior interaction can be destabilised in large populations as it may become harder to keep track of and remember past interactions and reputations (Boyd & Richerson, 1988; Milinski et al., 2001). There is empirical evidence that demographic and ecological factors, such as household organisation, population size and age structure, geography and environmental productivity, affect cooperation across human populations (Blurton Jones, 2016; Gurven et al., 2002; Lamba & Mace, 2011, 2013)

##### Hypotheses for microfinance research

Repayment efficacy may be structured by socio-cultural group if there are similarities in ecology and demography associated with these socio-cultural group boundaries. Repayment efficacy may be structured at any level at which ecology and demography are shared, i.e. community, village, ethno-linguistic group, any other kind of interaction group. Specifically, JLL groups of the same socio-cultural group (e.g. whose members were born or live in the same area) should have more similar repayment rates to groups from a different socio-cultural group, after controlling for other differences. Additionally, demographic factors such as population size and rates of migration might be associated with repayment efficacy. For example, repayment efficacy might be higher in smaller populations and populations with less migration (e.g. rural rather than urban populations).

### 3.3. Summary and clarifying comments

In the previous sections we have described several evolutionary mechanisms by which cooperators may preferentially assort (common ancestry, prior interaction, partner choice, similarity of tags, social learning, and ecology and demography), thus leading to the maintenance of cooperation. For each of these, we have specified hypotheses for how the mechanism may affect repayment efficacy in JLL-based microfinance loan groups. Before testing some of these hypotheses in the existing microfinance literature in Section 4, we offer some clarifications of these hypotheses, as well as of evolutionary approaches to human behaviour more generally, with a social science audience in mind.

First, it is important to keep in mind that these mechanisms may not be mutually exclusive (Sachs et al., 2004), i.e. multiple mechanisms may operate simultaneously in the same loan group. For example, people may select loan group members (partner choice) partly based on their reputation (prior interaction).

Second, while some of these mechanisms rely on a genetic basis for cooperative behaviour (e.g. common ancestry, see Section 3.2.1), other mechanisms assume that behaviour is acquired and transmitted nongenetically (e.g. via social learning, see Section 3.2.5). Therefore, an evolutionary approach does not require that cooperative behaviour itself is entirely genetically determined, rather genetically evolved cognitive or psychological faculties such as memory or language may facilitate the ability to use these assortment mechanisms.

Third, we can therefore make a distinction between the assortment mechanism that is ultimately supporting the maintenance of cooperation (e.g. common ancestry) and the proximate physiological, developmental and psychological processes (e.g. kin recognition) by which that mechanism operates. This distinction between *ultimate* and *proximate* causes of behaviour (Tinbergen, 1963) is sometimes blurred in the social science literature, particularly relating to human cooperation (Alexander, 1987; West et al., 2007a). In group lending, for example, proximate motivations and emotions like fear (Dube & Kamath, 2018), trust (van Bastelaer & Leathers, 2006) or a ‘preference’ for others’ payoffs (Carpenter & Williams, 2014) do not distinguish between the ultimate assortment mechanisms at play. People may ‘trust’ both their kin and repeated interaction partners, but this does not explain why trust is directed towards these particular categories of people. Identifying the ultimate assortment mechanisms underlying human cooperation can allow us to make novel predictions about what characteristics of individuals and groups are likely to be associated with repayment efficacy.

Finally, evolutionary theory does not require that individuals are consciously aware of the ultimate evolutionary reason why they are performing a particular behaviour (Nettle et al., 2013). Indeed, people might more often cite proximate motivations and emotions such as fear, trust or love for why they may or may not cooperate with another person. Consequently, people's actual behaviour provides the appropriate data for testing our hypotheses rather than their self-reported motivations.

## 4. Empirical review: do predictors of loan repayment correspond to evolutionary mechanisms of cooperation?

In this section we attempt to test the first three assortment mechanisms presented in Section 3.2, common ancestry, prior interaction and partner choice by examining the empirical literature. At this stage, we do not explicitly test the other three mechanisms – similarity of tags, social learning, and ecology and demography. These mechanisms cannot be tested by collecting simple associations between predictors and repayment outcomes from separated contexts. Rather, they require the examination of patterns of relative variation in a multilevel dataset, for example with borrowers nested within loan groups, nested within MFIs, nested within socio-cultural groups, nested within regions, etc. (see, e.g. Lamba & Mace, 2011), to identify which levels best structure the variance in repayment efficacy (see Sections 3.2.4–3.2.6). Furthermore, these latter three mechanisms overlap in their predictions. For example, all three mechanisms predict that repayment efficacy may be structured by socio-cultural group boundaries or tags. Multilevel data are essential to distinguish between them. This analytical approach is beyond the scope of this initial review. Here, we simply assess whether the empirical MFI literature contains predictors that can be related to evolutionary assortment mechanisms and conduct a rough assessment of the evidence for only the first three (common ancestry, prior interaction and partner choice).

Economists and other social scientists have conducted multiple empirical studies exploring the factors linked to the repayment efficacy of microfinance groups. Their aim is typically to assess what mitigates the incentive problems inherent in microfinance, such as the temptation to dishonestly misuse the loan (‘moral hazard’) or to evade the repayment obligation that is difficult to enforce (Hermes & Lensink, 2007). These studies compare multiple loan groups, usually from the same MFI and area, to examine whether certain characteristics of loan groups or borrowers are associated with higher or lower repayment efficacy. Our first step was to review the MFI literature to identify factors that had been tested by economists and other social scientists for their association with repayment efficacy of JLL groups and that could plausibly be linked to our evolutionary mechanisms specified in Section 3.2, especially common ancestry, prior interaction and partner choice. Since these studies were not guided by an evolutionary framework, they do not consistently and precisely measure the evolutionary factors that we predict to be associated with cooperation. For example, some studies measure kinship within the group as number of family members within the loan group, while others measure the percentage of group members related to each other. Consequently, we grouped factors from the literature into categories that themselves fell under the first three assortment mechanisms. For example, several authors measured associations between some form of geographical proximity between loan group members and repayment efficacy. We therefore created a category ‘geographical proximity’ to include these associations, which is likely to reflect the assortment mechanism of prior interaction.

We conduct a qualitative ‘vote count’ of findings within each category based on statistical significance (Koricheva et al., 2013). We count the number of associations that were in line with the evolutionary hypothesis (‘positive’), not in line (‘negative’) and that showed no statistical significance (‘nonsignificant’). We acknowledge that the ‘vote-counting’ methodology has clear weaknesses: it does not properly account for the size and uncertainty of effects, relies on results from error-prone statistical null hypothesis testing and weighs equally studies with different sample sizes, methods and quality (Koricheva et al., 2013). Further, despite our systematic approach, we cannot claim that this review is exhaustive or unbiased, since the collection of literature and effects involved multiple steps that were exploratory. Nevertheless, it provides a first systematic overview of the MFI literature within an evolutionary framework. A more rigorous quantitative meta-analysis would be the next step, but is challenging given the heterogeneity of the microfinance literature (Duvendack et al., 2011). Therefore, here we first assess the feasibility of pursuing this research agenda.

### 4.1. Review methodology

#### 4.1.1. Literature search

We searched Google Scholar for various combinations of the terms: ‘microfinance’, ‘microcredit’, ‘group lending’, ‘joint liability’, ‘repayment’, ‘default’, ‘delinquency’, ‘arrears’, ‘cooperation’, ‘social capital’, ‘factors’, ‘determinants’, ‘predictors’. We first screened for studies in the peer-reviewed and grey literature (dissertations, working papers, preprints) that (a) analysed factors associated with a measure of repayment efficacy (e.g. timeliness of repayment, proportion of loan repaid at due date, full loan repaid yes/no, occurrence of repayment problems yes/no) in groups that take out loans and have some degree of joint liability, (b) were quantitative and (c) reported methods and results sufficiently. We further screened the resulting papers to exclude studies that (a) reported aggregate data at the level of an entire country or MFI rather than at the level of individual borrowers or loan groups, (b) used lab or lab-in-the-field experiments (Harrison & List, 2004) on MFIs instead of data from real MFIs and (c) did not report results for a predictor that links to the evolutionary hypotheses we set out to examine (Section 3.2). Thus, we built a database of predictors examined in the MFI literature that link to the evolutionary hypotheses we wish to test (Section 3.2). Building on Lamba (2014), we focus on a set of predictors which is by no means an exhaustive list of all predictors occurring in the literature. Since no other previous work has been guided by similar hypotheses, we invite readers to examine our choices in the Supplementary Material 1 (SM1) and build upon them. We also scanned the bibliographies of all included work to find additional relevant studies. Based on this, predictors from a set of 41 studies were included in this review, with most of them published in economics and development studies journals (see SM1 for a full overview).

#### 4.1.2. Extraction of effects of predictors on repayment efficacy from reviewed literature

For each predictor variable included in our database we qualitatively recorded whether their association with repayment efficacy was statistically positive, negative or nonsignificant. We then recoded the predictors used in the original studies such that a positive association is in line with the evolutionary hypothesis, i.e. supports the hypothesis of a positive relationship between the evolutionary predictor and repayment efficacy, and negative associations are contrary to the evolutionary prediction, i.e. in the opposite direction. For example, we predict a *positive* relationship between geographical *proximity* and repayment efficacy (see Section 3.2.2). If a study reported a statistically significant *positive* association between ‘geographical *distance* between group members’ and repayment efficacy, we recoded the predictor as ‘geographical *proximity*’, and consequently recoded its relationship with repayment efficacy as *negative*. Therefore, since in this case ‘geographical *proximity*’ has a negative association with repayment efficacy, it is recorded as not in line with our evolutionary hypothesis. Note that some studies measured repayment efficacy inversely (e.g. credit default or repayment delay, see SM1); however we never recoded this in SM1, only the predictors.

The microfinance literature includes both characteristics of borrowers and characteristics of loan groups as predictors of repayment efficacy. We have specified in our database in SM1 the level at which the predictor was specified by referring to borrower or loan group as appropriate in the description of the predictor.

We did not include predictor variables measuring psychological states such as trust, anger, a willingness to exert pressure on others or self-reported motives for repayment (e.g. Dube & Kamath, 2018; Griffin & Husted, 2015), because these do not directly capture assortment mechanisms for the evolution of cooperation. Rather, these represent proximate psychological motivations (see Section 3.3) which were not the focus of this study.

When multiple analyses were reported for the same predictors in one study (e.g. various types of models and/or varying sets of covariates were reported), we only extracted *one association per predictor per study*. We selected only the analysis explicitly favoured by the original authors, except if there was a regression model with even more predictors included simultaneously, i.e. a ‘full model’. In that case, we selected the association reported in the larger model. In rare cases, when the analysis favoured by the original authors was not judged appropriate for the purpose of this review, the most suitable analysis was chosen by the first author (SG). For example, Griffin and Husted (2015) aggregated different questionnaire items into one latent variable, but the raw correlation between the questionnaire item of interest with repayment efficacy was seen as more suitable. When multiple measures for repayment efficacy were reported, we always selected whichever was most conservative in classifying repayment as low. For example, when both repayment problems within the loan group, i.e. individual borrowers not paying their shares (‘internal delinquency’), and repayment problems between the loan group and the MFI, i.e. the loan group not repaying its collective loan (‘external delinquency’), were reported, we used the latter. We invite readers to assess our choices of extracted variables, models and effects reported in SM1. We report associations as positive, negative or nonsignificant in accordance with the original authors’ own most permissive significance cut-offs (*p* < 0.1 in the majority of studies; SM1). In all studies, only monotonic relationships between predictors and repayment efficacy were analysed, except for two studies (Kolstad et al., 2017; Sabin & Reed-Tsochas, 2018), which reported an inverted U-shaped relationship between a predictor and repayment efficacy. Thus, in the results, ‘Inverted U’ is reported as an extra category.

#### 4.1.3. Categorisation of predictors

**Figure 2.**
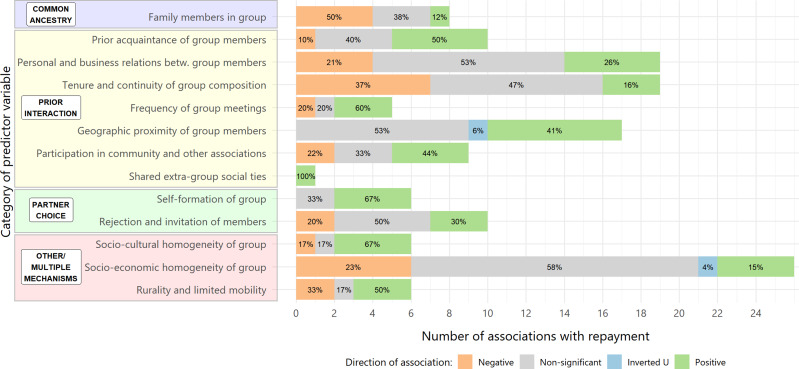
Number and qualitative directions of associations between predictor categories and repayment efficacy of JLL borrowers reported in the literature. Predictors are grouped into 13 categories based on the concept they are measuring. Coloured boxes further group these 13 categories into the evolutionary assortment mechanisms (see Section 4.1.3 for details). Associations are reported qualitatively as negative (orange), nonsignificant (grey), inverted U-shaped (blue) or positive (green) as per the original study (see Section 4.1.2 for details) with percentages of each type of association shown. Note that some bars do not add up exactly to 100% owing to rounding. The figure is based on a total of 142 associations from 41 empirical studies spanning 32 countries. See SM1 for details and references for every association extracted from the literature and SM2 for a table with the data underlying this figure. Within the group of categories labelled ‘Prior interaction’, the first six categories relate to direct reciprocity, and the last three to indirect reciprocity, with two categories relating to both direct or indirect reciprocity, specifically ‘Geographic proximity of group members’ and ‘Participation in community and other associations’. ‘Geographic proximity of group members’ may also relate to the ‘Other/multiple mechanisms’ grouping.

After extracting predictors from the empirical microfinance literature, we grouped together predictors that measured the same underlying concept. This was necessary because different authors measured the same concept in different ways. For example, the predictors ‘Number of family members in group’ (Carpenter & Williams, 2014) and ‘Proportion of group members who have a close relative in the group’ (Ahlin & Townsend, 2007) from two different studies are both classified as ‘Family members in group’. Each predictor was only included in one category. This gave rise to 13 categories of predictors which are displayed in Figure 2. SM1 provides the original definition of all underlying predictor and outcome variables included in each category.

As shown in Figure 2, we link 10 of these 13 categories to one of the three assortment mechanisms (common ancestry, prior interaction, partner choice) that we test, and group the final three categories as ‘Other/multiple mechanisms’ because these categories relate to and overlap across the three assortment mechanisms (similarity of tags, social learning, ecology and demography) that we do not explicitly test. We group predictors into conceptually cohesive categories and then link these categories to assortment mechanisms, rather than directly assigning predictors to mechanisms, to capture the multiple ways in which the assortment mechanisms may work.

### 4.2. Results

In total, 142 associations from 41 studies covering 32 countries were included and classified into 13 broader categories, shown in Figure 2. We find 47 (33.1%) positive associations of the predictor and repayment, meaning that the evolutionary hypothesis is supported in these cases. Two associations (1.4%) are initially positive for low values of the predictors (spatial proximity of group members’ residences; household income similarity of group members), but then turn negative (inverted U-shaped, fit via a quadratic function). Thirty associations (21.1%) are negative. There are 63 (44.4%) nonsignificant associations. Thus, just under half of the associations are nonsignificant, about one-third are positive and one-fifth are negative.

The three assortment mechanisms that we test (common ancestry, prior interaction, partner choice) contain varying proportions of negative, nonsignificant and positive associations (Figure 2 and Table SM2). For common ancestry, 50% of associations are negative, 38% are nonsignificant and 12% are positive. For prior interaction, 19% of associations are negative, 45% are nonsignificant, 1% (one finding) are inverted U shaped and 35% are positive. For partner choice, 13% of associations are negative, 44% are nonsignificant and 44% are positive.

Each assortment mechanism comprises multiple categories of predictors that group together variables measuring the same concept across studies (Figure 2 and Table SM2). These categories of predictors also contain varying proportions of negative, nonsignificant and positive associations (Figure 2). For example, 10% of associations between ‘prior acquaintance of group members’ and repayment are negative, 40% are nonsignificant and 50% are positive. Meanwhile, 37% of associations between ‘tenure and continuity of group composition’ and repayment are negative, 47% are nonsignificant and 16% are positive. Proportions of negative, nonsignificant and positive associations for each of the 13 categories separately are reported in Figure 2 and SM2.

For the 30 studies that reported loan group size means or ranges, we calculated a mean group size of 9.83 borrowers, median group size of 9.15 borrowers and range of 2.6–35 borrowers (where ranges were reported we used the mid-point of the range for our calculations). The assessed predictor variables, outcome variables, types of analysis and other study characteristics are presented in SM1.

## 5. Discussion

### 5.1. Summary of findings

In this paper, we assess whether the empirical literature on repayment efficacy among microcredit loan groups contains predictors that can be related to evolutionary assortment mechanisms that maintain cooperation: common ancestry, prior interaction, partner choice, similarity of tags, social learning, and ecology and demography. We find several predictors that can be related to these mechanisms, the majority of which relate to prior interaction. We conduct a rough assessment of the evidence for three of these (common ancestry, prior interaction, partner choice). Overall, the majority of associations are statistically nonsignificant, about one-third are positive (in line with our evolutionary hypotheses), about one-fifth are negative (not in line with our evolutionary hypotheses), and two associations take an inverted-U shape.

For the three mechanisms that we explicitly test, we find different levels of support. For common ancestry, 88% of studies show either negative (50%) or no associations (38%) between kin and repayment efficacy, leaving only 12% showing positive association (one finding), meaning that this evolutionary mechanism of assortment finds little support. For prior interaction, 64% of associations between a measure of acquaintanceship or interaction frequency between loan group members and repayment efficacy are either negative (19%) or nonsignificant (45%), with 35% positive, meaning we find greater support for this mechanism. However, prior interaction contains seven categories of predictors (see Figure 2), and there is variation in the level of support for each category. A majority of associations are positive for three categories of predictors (prior acquaintance of group members, 50%; frequency of group meetings, 60%; and shared extra-group social ties, 100% (one study)). On the other hand, a majority of associations are negative or nonsignificant for four categories (personal and business relations between group members, 74%; tenure and continuity of group composition, 84%; geographic proximity of group members, 53%; and participation in community and other associations, 55%), although for the latter two categories at least one-third of the associations are positive and these outnumber the negative associations. Consequently, the seven categories combined show mixed support for the mechanism of prior interaction. For partner choice, 57% of associations between a measure of whether loan group members chose any of their group members and repayment efficacy are either negative (13%) or nonsignificant (44%), with 44% positive, meaning we find the most support for this mechanism. This mechanism comprises two categories of predictors and a majority of associations are positive for one category of predictors (self-formation of group, 67%) with no negative associations at all. Some 70% of associations are negative (20%) or nonsignificant (50%) for the other category (rejection and invitation of members), although 30% are positive and outnumber the negative associations. Consequently, both categories together show some support for this assortment mechanism. In summary, of the three assortment mechanisms that we explicitly examine, we find little support for common ancestry, and more support for prior interaction and partner choice.

For the three mechanisms that we do not explicitly test (similarity of tags, social learning, ecology and demography), we nonetheless find predictors in the MFI literature to which they may be related, and which we grouped into three categories (socio-cultural homogeneity of group, socio-economic homogeneity of group and rurality and limited mobility). We find broad support for two of these categories (socio-cultural homogeneity of group, and rurality and limited mobility), with a majority of associations positive (67% and 50% respectively) and the number of positive associations equal to or greater than a half of all associations. For the category socio-economic homogeneity of group, we find a large number of associations (26), indicating the popularity of these predictors in the economic literature, but also the fact that this category includes many types of predictors. There is limited support for socio-economic homogeneity, with a majority of associations either negative (23%) or nonsignificant (58%), and more negative than positive (15%) associations. However, as discussed in Section 4, it is unclear how to link these categories of predictors to our evolutionary mechanisms given that all three mechanisms make predictions that overlap across these categories. In any case, these predictor–outcome associations cannot be used to test these mechanisms and multilevel data are required. Furthermore, these categories lump together many different kinds of homogeneity, and in many cases this homogeneity is measured in the original study using a composite index that combines several characteristics of borrowers. For example, similarity of ethnic markers, religion or caste are included in the category socio-cultural homogeneity of group and similarity of occupation, gender, age, income and education are included in the category socio-economic homogeneity of group. Therefore, these associations are difficult to interpret.

### 5.2. The negative effect of common ancestry

The finding that the majority of associations between kin and repayment efficacy are negative (i.e. not in line with our evolutionary hypothesis) is surprising given that many evolutionary studies in humans (Kurzban et al., 2015) and other species (Strassmann et al., 2011) have shown that help is preferentially directed towards kin. It is possible that the evolutionary theory of kin selection fails to explain repayment behaviour in the context of microfinance. Alternatively, there are at least three potential reasons for this unexpected finding which are in fact consistent with the theory.

First, economists have suggested that enforcement of loan repayment may be difficult among relatives or that relatives may collude against the MFI (Ahlin & Townsend, 2007; Hermes & Lensink, 2007; Sharma and Zeller, 1997). This may nevertheless be consistent with kin selection if group members who are relatives do not want to impose costs on their kin by punishing them for nonrepayment or are more lenient in giving them more time to repay their loans, and if they are willing to bear the penalties of nonrepayment imposed by the lender. In fact, some MFIs like Akhuwat in Pakistan have entirely banned family members from joining the same loan group (Mahmud, 2019).

Second, if the loan group is embedded in a wider social network such that individuals have kin outside the loan group as well as inside, then the relationships between kin outside the loan group may influence the relationships between kin inside the loan group. For example, if one has lots of close relatives within one's village but one forms a loan group with more distant relatives, then if one's close kin in the village need help, one may help them at the expense of being able to repay one's share of the loan on time. In this case, although one is defaulting on kin within the loan group, it is to help closer kin outside the loan group, consistent with kin selection. For example, Cieslik et al. (2019) report how borrowers in Burundi prioritise benefitting their family and friends over compliance with MFI rules by spending loans in unruly ways (e.g. consumption spending) that negatively affect repayment but may increase the status of their family (e.g. by spending on social events) or deepen close social bonds (e.g. by spending on gifts).

Third, if the density of kin in the wider social network is high, then upon forming a loan group with some of these kin, one may prioritise one's own earnings over the earnings of one's kin because one is also in economic competition with those kin. For example, if a borrower lives in a small village and forms a loan group with her sister, and they both own shops which compete for the same business, then the borrower may invest in her own business at the expense of repaying her share of the loan. This phenomenon, where cooperation between kin is undermined by competition between them, has been described by evolutionary biologists as kin competition (Taylor, 1992; West et al., 2002). Interestingly, one of the studies in our dataset may provide potential evidence for this: when splitting up their sample, Ahlin and Townsend (2007) find a negative effect of kinship on loan repayment only in the more rural region of Thailand, where people might reside, and hence compete, more in extended families.

To summarise, two out of three reasons why kin has a negative effect on repayment may actually be in line with kin selection, while the third is consistent with another theory from evolutionary biology, kin competition. Thus, MFI loan groups demonstrate that kin selection need not always result in a positive association between kin and cooperation within an interaction group, for example when the wider social context is taken into account or when enforcement facilitates cooperation.

The observation that kinship can generate collusion (i.e. cooperation) within the loan group against the MFI suggests that other factors which increase within-group cohesion may similarly reduce repayment efficacy. For example, a decrease in repayment was observed in a microcredit programme in Sierra Leone when groups were ‘too cohesive’, measured by their geographical proximity (Sabin & Reed-Tsochas, 2018). When penalties by the lender are too small, cooperation for repayment at the loan-group level may be less relevant than cooperation at other levels, such as the household, neighbourhood, community or dyad. Frameworks of ‘linked games’ (Spagnolo, 1999) and multilevel selection (J. Henrich, 2004; Richerson et al., 2016; Waring et al., 2015) which address interconnected and nested social dilemmas can potentially help to understand these phenomena.

### 5.3. Limitations

The reviewed studies are diverse in the country/regions where they were conducted, in the methodology used, in the measurement of variables and in the level of empirical investigation (individual borrowers versus loan groups). Some authors interviewed multiple group members, others just one. Sometimes the repayment outcome was a more direct measure from administrative records (e.g. percentage of loan value not repaid at a certain date), while other studies measured more indirect outcomes via self-report (e.g. whether group members reported experiencing repayment difficulties). The same concepts were measured in different ways across studies and different sets of covariates were included in the statistical models across studies. Sample sizes and sizes of loan groups varied widely (SM1). We expect some false positives, owing to the multiple analyses run and variables tested within the same study, and false negatives, owing to low statistical power in many studies, in addition to potential publication bias. A quantitative meta-analysis with more rigorous inclusion criteria is an important next step.

Most of the associations in our database are cross-sectional and correlational, casting doubt on the causal relationship between predictor and repayment efficacy. For example, borrower groups formed via self-selection will probably include members that have stronger reciprocal ties than groups that were formed exogenously. Mahmud (2019) found that the more time people had to form their groups in one Pakistani MFI, the more the groups were composed of people who knew each other previously. This confounds prior interaction and partner choice which we have presented as separate mechanisms in Section 3.2. Selection bias may limit findings in cases where loans are only offered to, or taken up by, particular kinds of individuals (e.g. women). Reversed causality may occur, for example when repayment outcomes affect the social ties between borrowers, or when individuals attend group meetings more regularly because they have repayment difficulties, rather than vice versa (discussed by van Bastelaer & Leathers, 2006). Encouragingly, the few studies that attempt to explicitly test causality using quasi-experimental approaches (listed in SM1) find effects that are in line with our hypotheses: repeated interactions in group meetings (Dalla Pellegrina et al., 2017) and geographical proximity (Karlan, 2007), both led to higher repayment efficacy. In the future, natural experiments, longitudinal data and lab-in-the-field experiments could provide more robust tests of our evolutionary hypotheses.

Some positives associations in our review might plausibly be explained by factors other than assortment. As outlined in the introduction (Section 3.1), the evolution of cooperation also depends on the relative costs and benefits. For example, longer tenure of a group might increase assortment by facilitating repeated interactions among members (Section 3.2.2), but it may also improve the borrowers’ finances, thereby reducing the need for a microloan (Paxton et al., 2000) or cause borrowers to exhaust productive business opportunities in which they could invest their loan (Guttman, 2007). This reduces the individual benefits of disciplined repayment and loan renewal. In terms of Hamilton's rule (Section 3.1), in this case though *r* may increase, *b* may simultaneously decrease. This could explain why this category of predictors (‘tenure and continuity of group composition’, Figure 2) has fewer positive associations with repayment than other proxies for prior interaction.

### 5.4. Implications for evolutionary biology and economics

We end by noting some general implications of our study. First, our unexpected finding that kin in the loan group are negatively associated with repayment efficacy highlights the need for evolutionary biologists and evolutionary human scientists to go beyond simplistic applications of evolutionary theory that make general claims that cooperation should always be preferentially directed towards kin, and test more nuanced predictions that arise from kin selection theory. Conversely, this also offers novel avenues of investigation for economists and social scientists studying microfinance. For example, the direction of the effect of kin on repayment efficacy may depend on the degree of kinship not only within the loan group, but also outside the group in the wider population. Typically, only the former is measured in microfinance studies.

Second, it is important to think about the overall structure of the cooperative dilemma (‘game’) that joint liability borrowers face as cooperative dilemmas come in different forms (Archetti & Scheuring, 2011, 2012). Once this is more clearly defined, predictions for different levels of assortment on the evolution of cooperation in this particular dilemma can be derived more precisely. A plausible model of JLL is a threshold public goods game: if a group's credit is fully repaid (i.e. the threshold is reached), all group members are rewarded with a new, larger loan, but under a strict implementation of JLL the lender is indifferent to who repays. Such dilemmas have been theoretically examined in economics (e.g. Palfrey & Rosenthal, 1984) and have been studied under the name of the ‘volunteer's dilemma’ in sociology (Diekmann, 1985) and biology (Archetti, 2009). Indeed, theoretical work suggests that it is easier to maintain cooperation in a threshold public goods game than in a linear public goods game, and that the role of assortment may be less crucial (Archetti, 2009; Archetti & Scheuring, 2011). This is generally consistent with the high repayment efficacy among group lending programmes globally.

Third, economists acknowledge the important role of different forms of ‘social capital’ for loan repayment in the microfinance literature (Armendáriz & Morduch, 2010; Cassar et al., 2007; Hermes & Lensink, 2007). Coleman (1988) defines social capital as social structure which facilitates particular actions of actors within that structure. In his definition, Coleman specifically highlights the roles of mutual obligation, expectations, trustworthiness, social norms, social sanctions and the transmission of information. In the context of JLL, social capital thus defined appears to comprise mechanisms that facilitate cooperators to selectively interact with each other. We propose that these are the assortment mechanisms described in Section 3.2, leading to the maintenance of cooperation. Therefore *r*, i.e. the degree of assortment in Hamilton's equation (Section 3.1), can be interpreted as a general measure of the effects of social capital. However, whereas the evolutionary biology literature distinguishes between ultimate causes of behaviour and the proximate motivations and preferences that support them (Section 3.3), the term social capital as used and measured by economists does not make this distinction. By distinguishing the two, an evolutionary approach allows us to identify the ultimate reasons why a behaviour or relationship occurs. For example, it allows us to explain *why* trust is directed at certain individuals or *why* certain peer networks are more or less conducive to successful repayment. This unpacking of social capital may lead to new insights and novel predictions in economic research. The concept of social capital emphasises not just the relations between actors but also the social structure in which they are embedded. This may serve as a reminder to the evolutionary sciences that measurements of *r* might benefit from taking account of not only the relatedness or degree of assortment of individuals within an interaction group but also the relative degrees of assortment within and between groups (see discussion Section 5.2).

In conclusion, we hope to have demonstrated the feasibility of using microfinance as a real-world test case for theories of the evolution of cooperation, and conversely that evolutionary theory may provide a unifying framework for organising existing findings on microfinance and generating new predictions regarding loan repayment under joint liability.

## Data Availability

R code and results from the literature review to reproduce Figure 2 and Table SM2 are available at: https://github.com/stefgehrig/bankingoncooperation.

## Supplementary material

For supplementary material accompanying this paper visit https://doi.org/10.1017/ehs.2020.64. click here to view supplementary material
