# Supplementary material for: Banking on cooperation: an evolutionary analysis of microfinance loan repayment behaviour
Source: Evol Hum Sci. 2020 Dec 14;3:e2. doi: 10.1017/ehs.2020.64 (PMC10427283; doi:10.1017/ehs.2020.64)
Supplement: Supplementary file 1 [file S2513843X2000064Xsup001.pdf]

Supplementary Material for “Banking on cooperation: An evolutionary analysis of microfinance loan repayment behaviour”

Stefan Gehrig, Alex Mesoudi, Shakti Lamba

Content

SM1: Details on included studies, predictors and associations from the microfinance literature 1

SM2: Summary of review results for all categories of predictors 19

Supplementary references 20

SM1: Details on included studies, predictors and associations from the microfinance literature

Table S1. Details on included studies, associations between predictors and repayment outcome and categorisation of predictors extracted for this review of the empirical literature on microfinance loan repayment under joint liability.

Category of predictor variable	**Predictor[a,b] (description of measured variable)**	**Repayment outcome[b] (description of measured variable)**	**Association with repayment outcome[c]**	**p-value[d]**	**No. of groups in sample[e]**	**Group size[f]**	**Country**	**Reference**	**Source and statistical model**	**Identification of causality[g]**
Family members in group[h]	Number of family members in group (continuous)	Occurrence of repayment problems (binary)	+	<0.05	≤135	2.6 (0.7)	Dominican Republic	(Matta, 2004)	Table 12, Logit model	None
	There are family relations in the group (binary)	Credits are repaid on time (binary)	ns	>0.10	6	14.3	Armenia	(Kasarjyan et al., 2007)	Table 2, Logit model	None
	Percentage of group members that are related to at least one other group member (continuous)	Number of days loans that were overdue in current loan cycle (continuous)	ns	>0.10	160	4-6	Jordan	(Al-Azzam et al., 2012)	Table 4 (column 1), Negative binomial model	None
	Number of family relations in group (continuous)	Group survival time (continuous)	ns	>0.10	1,064	15	India	(Baland et al., 2008)	Table 9, Weibull survival model	None
	Majority of group members are relatives (binary)	Number of group loans paid on time (continuous)	–	<0.05	120	Not reported	Ghana	(Bumbie, 2013)	Table 20, OLS model	None
	Percentage of group members related to each other (continuous)	Percentage of loan not repaid at due date (continuous)	–	<0.05	128	12.5	Bangladesh	(Sharma and Zeller, 1997)	Table 2, Tobit model	None
	Percentage of group members having a close relative in the group (continuous)	Group never been penalised for late repayment (binary)	–	<0.05	219	12.3 (5.1)	Thailand	(Ahlin and Townsend, 2007)	Table 3 (column 2), Logit model	None
	Number of family members in group (continuous)	Recorded repayment problems (binary)	–	<0.01	4	15-20	Paraguay	(Carpenter and Williams, 2014)	Table 6 (column 1), Probit model	None

Category of predictor variable	**Predictor[a,b] (description of measured variable)**	**Repayment outcome[b] (description of measured variable)**	**Association with repayment outcome[c]**	**p-value[d]**	**No. of groups in sample[e]**	**Group size[f]**	**Country**	**Reference**	**Source and statistical model**	**Identification of causality[g]**
Prior acquaintance of group members	Group members knew each other before the group was formed (binary)	All repayments on time (binary)	+	0.057	87	Not reported	Ethiopia	(Asgedom, 2015)	Table 6, Logit model	None
	Group existed previously for other purposes (binary)	Number of group loans paid on time (continuous)	+	<0.05	120	Not reported	Ghana	(Bumbie, 2013)	Table 20 (column 1), OLS model	None
	Group members knew each other before (binary)	Incidence of group delinquency (binary)	+	<0.05	286	4.1 (0.8)	Tunisia	(Bassem, 2008)	Table 2 (column 2), Logit model	None
	Majority of group members knew each other before forming the group on scale from 1-9 (continuous)	Punctuality of payments in last year on scale from 1-7 (continuous)	+	<0.01	182	5-12	Mexico	(Griffin and Husted, 2015)	Table 1, Spearman correlation	None
	Borrower knew group member from before the group was formed (binary)	Average number of missed payments for a group over the loan cycle (continuous)	+	<0.10	≤755	3-6	Pakistan	(Mahmud, 2019)	Table S5 (column 1), 2SLS model	Instrumental variable
	Group members had worked together in any other type of group (binary)	Incidence of loan arrears in group (binary)	ns	>0.05	140	5	Burkina Faso	(Paxton et al., 2000)	Table 2 (column 2), Nonmetric structural equation model	None
	At least two members knew each other before (binary)	% of loans overdue by greater than 30 days (continuous)	ns	>0.10	63	5.6 (4-10)	USA	(Anthony, 2005)	Table 3 (column 2), OLS model	None
	Number of years group members knew each other (continuous)	Group has average arrears of <3 days per loan and no loans in arrears >7 days (binary)	ns	>0.10	137	3.3 (0.5)	Guatemala	(Wydick, 1999)	Table 4 (column 3), Logit model	None
	Group members knew other group members before forming the group (binary)	Incidence of loan misuse (binary)	ns[*]	>0.10	102	4.5 (3-7)	Eritrea	(Hermes et al., 2005)	Table 4 (column 1), Logit model	None
	Number of years group members knew each other (continuous)	Occurrence of repayment problems (binary)	–	<0.01	≤135	2.6 (0.7)	Dominican Republic	(Matta, 2004)	Table 12, Logit model	None

Category of predictor variable	**Predictor[a,b] (description of measured variable)**	**Repayment outcome[b] (description of measured variable)**	**Association with repayment outcome[c]**	**p-value[d]**	**No. of groups in sample[e]**	**Group size[f]**	**Country**	**Reference**	**Source and statistical model**	**Identification of causality[g]**
Geographic proximity of group members	Percentage of group members within 10-minute walk from focal individual's home (continuous)	Percentage of loan in default at end of first cycle (continuous)	+	<0.01	42	15	Peru	(Karlan, 2007)	Table 4 (column 2), Tobit model	Natural experiment
	Percentage of group members living in same village (continuous)	Never been penalised for late repayment (binary)	+	<0.05	219	12.3 (5.1)	Thailand	(Ahlin and Townsend, 2007)	Table 3 (column 2), Logit model	None
	Average proximity between group members is 1 km or less (binary)	Number of days loans were overdue in current loan cycle (continuous)	+	<0.05	160	4-6	Jordan	(Al-Azzam et al., 2012)	Table 4 (column 1), Negative binomial model	None
	Average proximity of group members' businesses (continuous)	Group has average arrears of <3 days per loan and no loans in arrears >7 days (binary)	+	<0.05	137	3.3 (0.5)	Guatemala	(Wydick, 1999)	Table 4 (column 3), Logit model	None
	Group member is a neighbour measured for each borrower (binary)	Average number of missed payments for a group over the loan cycle measured for each borrower (continuous)	+	<0.10	≤755	3-6	Pakistan	(Mahmud, 2019)	Table S5 (column 3), 2SLS model	Instrumental variable
	Number of group members from same village (continuous)	Incidence of wilful default or misused loan (binary)	+	<0.01	99	16.8 (5.6)	Malawi	(Simtowe et al., 2006)	Table 6, Probit model	None
	Group members live in the same street or village (binary)	Every monthly payment is repaid on time (binary)	+	<0.01	493	Not reported	China	(Chen et al., 2020)	Table 2, Logit model	None
	Proximity of group members' residential addresses (continuous)	Number of days of longest payment delay occurring in group (continuous)	∩	<0.05	406	4.6 (0.5)	Sierra Leone	(Sabin and Reed-Tsochas, 2018)	Table 5 (column 2), OLS model	None
	Proximity of other group members' homes (continuous)	Percentage of loan in default at end of first cycle (continuous)	ns	>0.10	42	15	Peru	(Karlan, 2007)	Table 4 (column 2), Tobit model	Natural experiment
	Group member born in same area where survey was held (binary)	Incidence of loan misuse (binary)	ns	>0.10	102	4.5 (3-7)	Eritrea	(Hermes et al., 2005)	Table 4 (column 1), Logit model	None

Category of predictor variable	**Predictor[a,b] (description of measured variable)**	**Repayment outcome[b] (description of measured variable)**	**Association with repayment outcome[c]**	**p-value[d]**	**No. of groups in sample[e]**	**Group size[f]**	**Country**	**Reference**	**Source and statistical model**	**Identification of causality[g]**
Geographic proximity of group members	Proximity of group members (continuous)	Incidence of loan misuse (binary)	ns*	>0.10	102	4.5 (3-7)	Eritrea	(Hermes et al., 2005)	Table 4 (column 1), Logit model	None
	Members were born in the same area (binary)	All repayments on time (binary)	ns	0.865	87	Not reported	Ethiopia	(Asgedom, 2015)	Table 6, Logit model	None
	Number of years that borrower has lived in the current area (continuous)	Borrower reported repayment problems (binary)	ns	>0.10	289	11.1	Mexico	(Postelnicu et al., 2019)	Table 4 (fully reported in Supplementary Materials; column 1), Logit model	None
	Proximity of group members' houses (continuous)	Percentage of loan repaid (continuous)	ns	>0.15	118	24% of groups ≥12	Zambia	(van Bastelaer and Leathers, 2006)	Appendix A (column 4), OLS model	None
	Average proximity of other group members (continuous)	Incidence of group delinquency (binary)	ns	>0.10	286	4.1 (0.8)	Tunisia	(Bassem, 2008)	Table 2 (column 2), Logit model	None
	Average distance between group members is 1 km or less (binary)	Incidence of late repayment in last 9 months (binary)	ns	0.459	36	13.5	Togo	(Noglo and Androuais, 2015)	Table 2, Logit model	None
	Group members are from same district (binary)	Incidence of late repayment in last 9 months (binary)	ns	0.123	36	13.5	Togo	(Noglo and Androuais, 2015)	Table 2, Logit model	None

Category of predictor variable	**Predictor[a,b] (description of measured variable)**	**Repayment outcome[b] (description of measured variable)**	**Association with repayment outcome[c]**	**p-value[d]**	**No. of groups in sample[e]**	**Group size[f]**	**Country**	**Reference**	**Source and statistical model**	**Identification of causality[g]**
Personal and business relations between group members	Group members regularly visit each other (binary)	All repayments on time (binary)	+	0.048	87	Not reported	Ethiopia	(Asgedom, 2015)	Table 6, Logit model	None
	Group members have joint enterprise (binary)	Incidence of wilful default or misused loan (binary)	+	<0.10	99	16.8 (5.6)	Malawi	(Simtowe et al., 2006)	Table 6, Probit model	None
	Group members visit each other ≥7 times per month (binary)	Incidence of late repayment in last 9 months (binary)	+	<0.001	36	13.5	Togo	(Noglo and Androuais, 2015)	Table 2, Logit model	None
	Number of positive responses by group leader to 5 questions on help received by group leader from other group members in personal issues, e.g., child care, mediation (continuous)	Number of days groups' loans were overdue in current loan cycle (continuous)	+	<0.01	160	4-6	Jordan	(Al-Azzam et al., 2012)	Table 4 (column 1), Negative binomial model	None
	Number of positive responses at group level to two questions on whether members ever cooperated on personal or business matters (continuous)	Percentage of group loans overdue by more than 30 days (continuous)	+	<0.05	63	5.6 (4-10)	USA	(Anthony, 2005)	Table 3 (column 2), OLS model	None
	Group members are friends (binary)	Group has average arrears of <3 days per loan and no loans in arrears >7 days (binary)	ns	>0.10	137	3.3 (0.5)	Guatemala	(Wydick, 1999)	Table 4 (column 3), Logit model	None
	Group members take part in social activities together (binary)	Group has average arrears of <3 days per loan and no loans in arrears >7 days (binary)	ns	>0.10	137	3.3 (0.5)	Guatemala	(Wydick, 1999)	Table 4 (column 3), Logit model	None

Category of predictor variable	**Predictor[a,b] (description of measured variable)**	**Repayment outcome[b] (description of measured variable)**	**Association with repayment outcome[c]**	**p-value[d]**	**No. of groups in sample[e]**	**Group size[f]**	**Country**	**Reference**	**Source and statistical model**	**Identification of causality[g]**
Personal and business relations between group members	Borrower's number of ties with group members which are strong, with strength defined by two-means cluster analysis of, e.g., duration, geographical proximity, closeness, sharing in relationship (continuous)	Borrower reported repayment problems (binary)	ns	>0.10	289	11.1	Mexico	(Postelnicu et al., 2019)	Table 4 (fully reported in Supplementary materials; column 1), Logit model	None
	Number of positive responses by borrower to 7 questions on helping and closeness of group members, e.g. visiting them, buying products, soliciting advice (continuous)	Percentage of borrower's loan in default at due date (continuous)	ns	>0.10	≥844	5 (uniform)	Phillipines	(Giné and Karlan, 2014)	Table 8 (column 10), OLS model	None
	Number of friends in group (continuous)	Recorded repayment problems (binary)	ns	>0.10	4	15-20	Paraguay	(Carpenter and Williams, 2014)	Table 6 (column 1), Probit model	None
	Group members visit each other (binary)	Incidence of group delinquency (binary)	ns	>0.10	286	4.1 (0.8)	Tunisia	(Bassem, 2008)	Table 2 (column 2), Logit model	None
	Regular visits to group members (binary)	Incidence of loan misuse (binary)	ns	>0.10	102	4.5 (3-7)	Eritrea	(Hermes et al., 2005)	Table 4 (column 1), Logit model	None
	Frequency of contact between group members on scale from 0, no contact, to 4, more than once a week (continuous)	Incidence of non-repayment for ≥120 days (binary)	ns	>0.10	150	6 (4-8)	USA	(Anthony and Horne, 2003)	Table 3 (column 6), Logit model	None

Category of predictor variable	Predictor[a,b] (description of measured variable)	Repayment outcome[b] (description of measured variable)	Association with repayment outcome[c]	p-value[d]	No. of groups in sample[e]	Group size[f]	Country	Reference	Source and statistical model	Identification of causality[g]
Personal and business relations between group members	Seek help from group members for dispute mediation (binary)	Number of group loans paid on time (continuous)	ns	>0.10	120	Not reported	Ghana	(Bumbie, 2013)	Table 20 (column 1), OLS model	None
	Number of positive responses at group level to 3 questions on whether business decisions on crop, fertiliser and production technology were taken collectively (continuous)	Group has never been penalised for late repayment (binary)	ns	>0.15	219	12.3 (5.1)	Thailand	(Ahlin and Townsend, 2007)	Table 3 (column 2), Logit model	None
	Group members visit the same church (binary)	Percentage of loan repaid (binary)	–	<0.05	118	24 % of groups ≥12	Zambia	(van Bastelaer and Leathers, 2006)	Appendix A (column 4), OLS model	None
	Number of positive group level responses to 5 questions on helping amongst group members with labour, sales coordination, money etc. (continuous)	Group has never been penalised for late repayment (binary)	–	<0.05	219	12.3 (5.1)	Thailand	(Ahlin and Townsend, 2007)	Table 3 (column 2), Logit model	None
	Group members know the quality of each other's work (binary)	Never been penalised for late repayment (binary)	–	<0.15	219	12.3 (5.1)	Thailand	(Ahlin and Townsend, 2007)	Table 3 (column 2), Logit model	None
	Number of positive responses by group leader to 6 questions on helping between group members in business issues, e.g., referrals, free labour (continuous)	Number of days groups' loans were overdue in current loan cycle (continuous)	–	<0.05	160	4-6	Jordan	(Al-Azzam et al., 2012)	Table 4 (column 1), Negative binomial model	None

Category of predictor variable	**Predictor[a,b] (description of measured variable)**	**Repayment outcome[b] (description of measured variable)**	**Association with repayment outcome[c]**	**p-value[d]**	**No. of groups in sample[e]**	**Group size[f]**	**Country**	**Reference**	**Source and statistical model**	**Identification of causality[g]**
Tenure and continuity of group composition	Number of years group has been in operation (continuous)	Percentage of loan repaid (binary)	+	<0.15	118	24 % of groups ≥12	Zambia	(van Bastelaer and Leathers, 2006)	Appendix A (column 4), OLS model	None
	Number of original members still in the group (continuous)	Incidence of wilful default or misused loan (binary)	+	<0.01	99	16.8 (5.6)	Malawi	(Simtowe et al., 2006)	Table 6, Probit model	None
	Number of years the group has participated in the credit programme (continuous)	Loan is fully repaid by group (binary)	+	<0.001	150	Not reported	Tanzania	(Kinyondo and Okurut, 2009)	Table 1, Logit model	None
	Number of months the group has existed (continuous)	Percentage of loans overdue by more than 30 days (continuous)	ns	<0.01	63	5.6 (4-10)	USA	(Anthony, 2005)	Table 3 (column 2), OLS model	None
	Group has all its original members (binary)	Percentage of loan repaid (continuous)	ns	>0.15	118	24 % of groups ≥12	Zambia	(van Bastelaer and Leathers, 2006)	Appendix A (column 4), OLS model	None
	Number of years borrowing group has existed (continuous)	Percentage of loans overdue by greater than 30 days (continuous)	ns	>0.10	150	6 (4-8)	USA	(Anthony and Horne, 2003)	Table 3 (column 6), Logit model	None
	Age of borrower's group (continuous)	Borrower reported repayment problems (binary)	ns	>0.10	289	11.1	Mexico	(Postelnicu et al., 2019)	Table 4 (fully reported in Supplementary materials; column 1), Logit model	None
	Number of years group has existed (continuous)	Percentage of loan in default	ns	0.52	102	19 (15-30)	Kenya	(Amwayi et al., 2014)	Table 4.2, OLS model	None
	Number of years since group took first loan (continuous)	Incidence of late repayment in last 9 months (binary)	ns	0.12	36	13.5	Togo	(Noglo and Androuais, 2015)	Table 2, Logit model	None
	Number of years since group took first loan (continuous)	Group has average arrears of <3 days per loan and no loans in arrears >7 days (binary)	ns	>0.10	137	3.3 (0.5)	Guatemala	(Wydick, 1999)	Table 4 (column 3), Logit model	None

Category of predictor variable	**Predictor[a,b] (description of measured variable)**	**Repayment outcome[b] (description of measured variable)**	**Association with repayment outcome[c]**	**p-value[d]**	**No. of groups in sample[e]**	**Group size[f]**	**Country**	**Reference**	**Source and statistical model**	**Identification of causality[g]**
Tenure and continuity of group composition	Current loan cycle number of the group (continuous)	Number of days of longest payment delay occurring in group (continuous)	ns	>0.05	406	4.6 (0.5)	Sierra Leone	(Sabin and Reed-Tsochas, 2018)	Table 5 (column 2), OLS model	None
	Number of years group existed (continuous)	Group could repay loan (binary)	ns	0.465	40	Not reported	Ghana	(Ayogyam et al., 2014)	Table 3, Logit model	None
	Period of time during which the group exists (continuous)	Every monthly payment is repaid on time (binary)	-	<0.01	493	Not reported	China	(Chen et al., 2020)	Table 2, Logit model	None
	Current loan cycle number of the group (continuous)	Incidence of wilful default or misused loan (binary)	–	<0.05	99	16.8 (5.6)	Malawi	(Simtowe et al., 2006)	Table 6, Probit model	None
	Number of months group has existed on due date (continuous)	Loan repaid at due date (binary)	–	<0.05	≤905	Not reported	Bangladesh	(Godquin, 2004)	Table 7 (column 5), Probit model	None
	Seniority of membership in group, i.e. a measure of how many loan cycles the individual has taken with the group (continuous)	Degree of arrears, i.e. estimate of degree to which loan was paid on time (continuous)	–	<0.05	≤1159	30-40 (loan center level)	Bangladesh	(Guttman, 2007)	Table 1, OLS model	None
	Current loan cycle number of the group (continuous)	Incidence of loan arrears in group (binary)	–	<0.05	140	5	Burkina Faso	(Paxton et al., 2000)	Table 2 (column 2), Nonmetric structural equation model	None
	Number of years group existed (continuous)	Percentage of loan repaid by group (continuous)	–	<0.01	90	9.8	Nigeria	(Olomola, 2002)	Table 5 (column 1), OLS model	None
	Number of months the group existed (continuous)	Incidence of group delinquency (binary)	–	<0.05	286	4.1 (0.8)	Tunisia	(Bassem, 2008)	Table 2 (column 2), Logit model	None

Category of predictor variable	**Predictor[a,b] (description of measured variable)**	**Repayment outcome[b] (description of measured variable)**	**Association with repayment outcome[c]**	**p-value[d]**	**No. of groups in sample[e]**	**Group size[f]**	**Country**	**Reference**	**Source and statistical model**	**Identification of causality[g]**
Frequency of group meetings	Frequency of group meetings bimonthly instead of monthly (binary)	Number of repayments with delay (continuous)	+	<0.01	262	5 (uniform)	South Africa	(Dalla Pellegrina et al., 2017)	Table 8.1 (column 2),	Propensity score matching + Difference-in-difference
	Individual borrower's participation in credit meetings (binary)	Borrower's ratio of amount of loan repaid to the total amount due (continuous)	+	0.079	20	Not reported	Kenya	(Sungwacha et al., 2014)	Results table, Tobit model	None
	Borrower's ratio of attended and scheduled meetings per month (continuous)	Amount repaid by borrower divided by total amount borrowed (continuous)	+	<0.001	29	Not reported	Kenya	(Kangogo et al., 2013)	Table 8, Tobit model	None
	Number of days group members meet per month (continuous)	Proportion of loan repaid at due date (continuous)	ns	>0.05	≤200	Not reported	Nigeria	(Oke et al., 2007)	Table 8, OLS model	None
	Frequency of group meetings on scale from 1 to 5 (continuous)	Percentage of loan repaid (continuous)	–	<0.15	118	24 % of groups ≥12	Zambia	(van Bastelaer and Leathers, 2006)	Appendix A (column 4), OLS model	None
Shared extra-group social ties	Borrower's number of indirect ties with group members by knowing the same person outside the group ("information channels") which are strong, with strength defined by two-means cluster analysis of, e.g., duration, geographical proximity, closeness, sharing in relationship (continuous)	Borrower reported repayment problems (binary)	+	<0.05	289	11.1	Mexico	(Postelnicu et al., 2019)	Table 4 (fully reported in Supplementary Materials; column 1), Logit model	None

Category of predictor variable	**Predictor[a,b] (description of measured variable)**	**Repayment outcome[b] (description of measured variable)**	**Association with repayment outcome[c]**	**p-value[d]**	**No. of groups in sample[e]**	**Group size[f]**	**Country**	**Reference**	**Source and statistical model**	**Identification of causality[g]**
Participation in community and other associations	Borrower is member of production cooperative (binary)	Borrower's credits are repaid on time (binary)	+	<0.01	6	14.3	Armenia	(Kasarjyan et al., 2007)	Table 2, Logit model	None
	Borrower is member of informal networks (binary)	Borrower reported repayment problems (binary)	+	<0.01	289	11.1	Mexico	(Postelnicu et al., 2019)	Table 4 (fully reported in Supplementary materials; column 1), Logit model	None
	Group members have been members of other loan groups (binary)	All repayments on time (binary)	+	0.054	87	Not reported	Ethiopia	(Asgedom, 2015)	Table 6, Logit model	None
	Borrower is a member of other cooperative societies (binary)	Proportion of loan repaid by borrower at due date (continuous)	+	<0.01	≤200	Not reported	Nigeria	(Oke et al., 2007)	Table 8, OLS model	None
	Borrower is member of political party (binary)	Borrower's credits are repaid on time (binary)	ns	>0.01	6	14.3	Armenia	(Kasarjyan et al., 2007)	Table 2, Logit model	None
	Number of community groups borrower belongs to (continuous)	Recorded repayment problems (binary)	ns	>0.10	4	15-20	Paraguay	(Carpenter and Williams, 2014)	Table 6 (column 1), Probit model	None
	Borrower is member of formal networks (binary)	Borrower reported repayment problems (binary)	ns	>0.10	289	11.1	Mexico	(Postelnicu et al., 2019)	Table 4 (fully reported in Supplementary materials; column 1), Logit model	None
	Number of other loan group memberships in the borrower's household (continuous)	Amount repaid by borrower divided by total amount borrowed (continuous)	–	0.008	29	Not reported	Kenya	(Kangogo et al., 2013)	Table 8, Tobit model	None
	Borrower often talks with friends and neighbours about community problems (binary)	Ratio of borrower's on time repayments to the number of repayments required in the current loan cycle (continuous)	–	<0.10	27	Not reported	Nicaragua	(Mason, 2011)	Table 20 (column 2), OLS model	None

Category of predictor variable	**Predictor[a,b] (description of measured variable)**	**Repayment outcome[b] (description of measured variable)**	**Association with repayment outcome[c]**	**p-value[d]**	**No. of groups in sample[e]**	**Group size[f]**	**Country**	**Reference**	**Source and statistical model**	**Identification of causality[g]**
Self-formation of group	Group formed on its own (binary)	Percentage of loan not repaid at due date (continuous)	+	<0.10	128	12.5	Bangladesh	(Sharma and Zeller, 1997)	Table 2, Tobit model	None
	Number of months (0-18) group had available for self-formation (continuous)	Average number of missed payments for a group over the loan cycle (continuous)	+	<0.10	755	3-6	Pakistan	(Mahmud, 2019)	Table 9 & 10 combined (2SLS model)	Natural experiment (this predictor is used to instrument for other predictors)
	Group formed by peer selection (binary)	Incidence of wilful default or misused loan (binary)	+	<0.10	99	16.8 (5.6)	Malawi	(Simtowe et al., 2006)	Table 6, Probit model	None
	Group formed by self-selection (binary)	Number of group loans paid on time (continuous)	+	<0.10	120	Not reported	Ghana	(Bumbie, 2013)	Table 20 (column 1), OLS model	None
	Borrower's group was initiated by borrowers (binary)	Borrower reported repayment problems (binary)	ns	>0.10	289	11.1	Mexico	(Postelnicu et al., 2019)	Table 4 (fully reported in Supplementary materials; column 1), Logit model	None
	Group initiated by group members rather than external agent (binary)	Percentage of loans fully repaid by the group on due date (continuous)	ns	>0.10	141	10.2 (4.8)	Madagascar	(Zeller, 1998)	Table 4, Tobit model	None

Category of predictor variable	**Predictor[a,b] (description of measured variable)**	**Repayment outcome[b] (description of measured variable)**	**Association with repayment outcome[c]**	**p-value[d]**	**No. of groups in sample[e]**	**Group size[f]**	**Country**	**Reference**	**Source and statistical model**	**Identification of causality[g]**
Rejection and invitation of members	Group ever rejected a candidate interested in joining (binary)	Number of group loans paid on time (continuous)	+	<0.05	120	Not reported	Ghana	(Bumbie, 2013)	Table 20 (column 1), OLS model	None
	Group rejects borrowers who want to join (binary)	Incidence of group delinquency (binary)	+	<0.01	286	4.1 (0.8)	Tunisia	(Bassem, 2008)	Table 2 (column 2), Logit model	None
	Percentage of group members knowing group composition before or after formation of group (continuous)	Incidence of wilful default or misused loan (binary)	+	<0.10	99	16.8 (5.6)	Malawi	(Simtowe et al., 2006)	Table 6, Probit model	None
	Group rejected individuals who wanted to join the group (binary)	Incidence of wilful default or misused loan (binary)	ns	>0.10	99	16.8 (5.6)	Malawi	(Simtowe et al., 2006)	Table 6, Probit model	None
	Borrower joined the loan group by invitation (binary)	Ratio of timely repayments to number of repayments required in the current loan cycle (continuous)	ns	>0.10	27	Not reported	Nicaragua	(Mason, 2011)	Table 20 (column 2), OLS model	None
	Group rejected a person who wanted to join (binary)	Number of days loans were overdue in current loan cycle (continuous)	ns	>0.10	160	4-6	Jordan	(Al-Azzam et al., 2012)	Table 4 (column 1), Negative binomial model	None
	Some individuals want to join the group but cannot (binary)	Never been penalised for late repayment (binary)	ns	>0.15	219	12.3 (5.1)	Thailand	(Ahlin and Townsend, 2007)	Table 3 (column 2), Logit model	None
	Group screened membership according to reputation (binary)	Loan not fully paid on due date (binary)	ns	>0.10	25	Not reported	Costa Rica	(Wenner, 1995)	Table 6 (column 3), Multinomial logit model	None
	Group ever rejected someone who wanted to join the group (binary)	Incidence of late repayment in last 9 months (binary)	–	<0.001	36	13.5	Togo	(Noglo and Androuais, 2015)	Table 2, Logit model	None
	Group ever rejected a borrower who would like to join the group (binary)	All repayments on time (binary)	–	0.060	87	Not reported	Ethiopia	(Asgedom, 2015)	Table 6, Logit model	None

Category of predictor variable	**Predictor[a,b] (description of measured variable)**	**Repayment outcome[b] (description of measured variable)**	**Association with repayment outcome[c]**	**p-value[d]**	**No. of groups in sample[e]**	**Group size[f]**	**Country**	**Reference**	**Source and statistical model**	**Identification of causality[g]**
Socio-cultural homogeneity of group	Percentage of group members with same cultural markers, e.g., language, hair, dress (continuous)	Percentage of loan in default at end of first cycle (continuous)	+	<0.01	42	15	Peru	(Karlan, 2007)	Table 4 (column 2), Tobit model	Natural experiment
	Group members are same ethnicity (binary)	Incidence of late repayment in last 9 months (binary)	+	<0.001	36	13.5	Togo	(Noglo and Androuais, 2015)	Table 2, Logit model	None
	Religious homogeneity within group (continuous)	Group could repay loan (binary)	+	0.052	40	Not reported	Ghana	(Ayogyam et al., 2014)	Table 3, Logit model	None
	Religious-linguistic homogeneity of group (continuous)	Group not active anymore (binary)	+	<0.05	51	10.6 (4.5)	Angola	(Kolstad et al., 2017)	Table 3 (column 5), OLS model	None
	Caste homogeneity of group (continuous)	Group survival time (continuous)	ns	>0.10	1,064	15	India	(Baland et al., 2008)	Table 9, Weibull survival model	None
	Group members are from same religion (binary)	Incidence of late repayment in last 9 months (binary)	–	<0.001	36	13.5	Togo	(Noglo and Androuais, 2015)	Table 2, Logit model	None
Socio-economic homogeneity of group	Number of common characteristics amongst group members in village or hamlet of origin, ethnicity, extended family, gender, religion (continuous)	Percentage of loans fully repaid by the group on due date (continuous)	+	<0.10	141	10.2 (4.8)	Madagascar	(Zeller, 1998)	Table 4, Tobit model	None
	Group members have same occupation (binary)	Incidence of late repayment in last 9 months (binary)	+	<0.001	36	13.5	Togo	(Noglo and Androuais, 2015)	Table 2, Logit model	None
	Group members have same gender (binary)	Incidence of late repayment in last 9 months (binary)	+	<0.001	36	13.5	Togo	(Noglo and Androuais, 2015)	Table 2, Logit model	None
	Group members have similar businesses (binary)	Every monthly payment is repaid on time (binary)	+	<0.01	493	Not reported	China	(Chen et al., 2020)	Table 2, Logit model	None
	Group members have same gender (binary)	Incidence of non-repayment for ≥120 days (binary)	ns	>0.10	150	6 (4-8)	USA	(Anthony and Horne, 2003)	Table 3 (column 6), Logit model	None

Category of predictor variable	**Predictor[a,b] (description of measured variable)**	**Repayment outcome[b] (description of measured variable)**	**Association with repayment outcome[c]**	**p-value[d]**	**No. of groups in sample[e]**	**Group size[f]**	**Country**	**Reference**	**Source and statistical model**	**Identification of causality[g]**
Socio-economic homogeneity of group	Group members have same gender (binary)	Group has average arrears of <3 days per loan and no loans in arrears >7 days (binary)	ns	>0.10	137	3.3 (0.5)	Guatemala	(Wydick, 1999)	Table 4 (column 3), Logit model	None
	Homogeneity in within-group household income (continuous)	Group not active anymore (binary)	∩	<0.05	51	10.6 (4.5)	Angola	(Kolstad et al., 2017)	Table 3 (column 5), OLS model	None
	Group members have same line of business (binary)	Group has average arrears of <3 days per loan and no loans in arrears >7 days (binary)	ns	>0.10	137	3.3 (0.5)	Guatemala	(Wydick, 1999)	Table 4 (column 3), Logit model	None
	Number of same business types within the group (continuous)	Number of days of longest payment delay occurring in group (continuous)	ns	>0.05	406	4.6 (0.5)	Sierra Leone	(Sabin and Reed-Tsochas, 2018)	Table 5 (column 2), OLS model	None
	Percentage of two randomly chosen group members having the same occupation (continuous)	Never been penalised for late repayment (binary)	ns	>0.15	219	12.3 (5.1)	Thailand	(Ahlin and Townsend, 2007)	Table 3 (column 2), Logit model	None
	Index (0-5) of group homogeneity including similarity of members' age cohort, wealth level, and social group, home proximity, business proximity (continuous)	Loan is fully repaid by group (binary)	ns	>0.10	150	Not reported	Tanzania	(Kinyondo and Okurut, 2009)	Table 1, Logit model	None

Category of predictor variable	**Predictor[a,b] (description of measured variable)**	**Repayment outcome[b] (description of measured variable)**	**Association with repayment outcome[c]**	**p-value[d]**	**No. of groups in sample[e]**	**Group size[f]**	**Country**	**Reference**	**Source and statistical model**	**Identification of causality[g]**
Socio-economic homogeneity of group	Index of borrower's group's homogeneity from 4 questions (on a 5-point scale) on similarity in economic background, proximity of business, proximity of home, frequency of communication (continuous)	Repayment by borrower of entire loan (binary)	ns	0.183	≤11	4-6	USA	(Bhatt and Tang, 2002)	Table 6, Logit model	None
	Sum of coefficients of variation in group members' age, gender, religion, ethnicity, nativity, civil status, residency, education, membership in other group (continuous)	Percentage of loan repaid by group (continuous)	ns	>0.10	90	9.8	Nigeria	(Olomola, 2002)	Table 5 (column 1), OLS model	None
	Sum of coefficients of variation in income and occupations of group (continuous)	Percentage of loan repaid by group (continuous)	ns	>0.10	90	9.8	Nigeria	(Olomola, 2002)	Table 5 (column 1), OLS model	None
	Group members have same education (binary)	Loan repaid at due date (binary)	ns	>0.10	≤905	Not reported	Bangladesh	(Godquin, 2004)	Table 7 (column 5), Probit model	None
	Group members have same age (binary)	Loan repaid at due date (binary)	ns	>0.10	≤905	Not reported	Bangladesh	(Godquin, 2004)	Table 7 (column 5), Probit model	None
	Number of common characteristics shared by group members in age, gender, education, income, place of living (continuous)	Repayment at due date by group (binary)	ns	>0.10	84	7-15	Georgia	(Kritikos and Vigenina, 2005)	Table 5 (column 1), Multinomial logit model	None
	Group members have same business (binary)	Incidence of group delinquency (binary)	ns	>0.10	286	4.1 (0.8)	Tunisia	(Bassem, 2008)	Table 2 (column 2), Logit model	None

Category of predictor variable	**Predictor[a,b] (description of measured variable)**	**Repayment outcome[b] (description of measured variable)**	**Association with repayment outcome[c]**	**p-value[d]**	**No. of groups in sample[e]**	**Group size[f]**	**Country**	**Reference**	**Source and statistical model**	**Identification of causality[g]**
Socio-economic homogeneity of group	Number of common characteristics within group in ethnicity, gender, age, income, activities, neighbourhood	Incidence of loan arrears in group (binary)	ns	>0.05	140	5	Burkina Faso	(Paxton et al., 2000)	Table 2 (column 2), Nonmetric structural equation model	None
	Percentage of group members starting business for same reason (continuous)	Percentage of loans overdue by greater than 30 days (continuous)	ns	>0.10	63	5.6 (4-10)	USA	(Anthony, 2005)	Table 3 (column 2), OLS model	None
	Group members have same gender (binary)	Incidence of loan misuse (binary)	–	<0.05	102	4.5 (3-7)	Eritrea	(Hermes et al., 2005)	Table 4 (column 1), Logit model	None
	Homogeneity of gender in group (unknown scaling)	Group could repay loan (binary)	–	0.030	40	Not reported	Ghana	(Ayogyam et al., 2014)	Table 3, Logit model	None
	Borrower's perception of group homogeneity in respect to income (binary)	Borrower's credits are repaid on time (binary)	–	<0.10	6	14.3	Armenia	(Kasarjyan et al., 2007)	Table 2, Logit model	None
	Group members are same age ± 2 years (binary)	Incidence of group delinquency (binary)	–	<0.01	286	4.1 (0.8)	Tunisia	(Bassem, 2008)	Table 2 (column 2), Logit model	None
	Group members are same gender (binary)	Incidence of group delinquency (binary)	–	<0.10	286	4.1 (0.8)	Tunisia	(Bassem, 2008)	Table 2 (column 2), Logit model	None
	Sum (0-5) of whether members are of same age group, same gender, same education level, same occupation and economic status (continuous)	Amount repaid divided by total amount borrowed (continuous)	–	0.004	29	Not reported	Kenya	(Kangogo et al., 2013)	Table 8, Tobit model	None

Category of predictor variable	**Predictor[a,b] (description of measured variable)**	**Repayment outcome[b] (description of measured variable)**	**Association with repayment outcome[c]**	**p-value[d]**	**No. of groups in sample[e]**	**Group size[f]**	**Country**	**Reference**	**Source and statistical model**	**Identification of causality[g]**
Rurality and limited mobility	Borrower's group is in rural area (binary)	Borrower reported repayment problems (binary)	+	<0.05	289	11.1	Mexico	(Postelnicu et al., 2019)	Table 4 (fully reported in Supplementary materials; column 1), Logit model	None
	Distance from group's village to infrastructural services (continuous)	Percentage of group loan not repaid at due date (continuous)	+	<0.10	128	12.5	Bangladesh	(Sharma and Zeller, 1997)	Table 2, Tobit model	None
	Lack of infrastructure available to group on scale from 0-5 (continuous)	Loan not fully paid on due date by group (binary)	+	<0.10	25	Not reported	Costa Rica	(Wenner, 1995)	Table 6 (column 3), Multinomial logit model	None
	Group is in rural area (binary)	Percentage of repayments made on time (continuous)	ns	>0.10	27	Not reported	Nicaragua	(Mason, 2011)	Table 20 (column 2), OLS model	None
	Group is in rural area (binary)	Incidence of loan arrears in group (binary)	–	<0.05	140	5	Burkina Faso	(Paxton et al., 2000)	Table 2 (column 2), Nonmetric structural equation model	None
	Average distance of group members from rail line (continuous)	Percentage of loan repaid by group (continuous)	–	<0.05	118	24 % of groups ≥12	Zambia	(van Bastelaer and Leathers, 2006)	Appendix A (column 4), OLS model	None

Notes: [a]Predictors are expressed in a way such that higher values refer to more expected cooperation based on the hypotheses in the main text. [b]In brackets we report whether the variable was measured as a binary category or as a continuous number. We also specify for each variable whether it was measured at the individual or loan group level, by referring to 'borrower / individual' or 'group' respectively where appropriate. [c]Associations are expressed such that + always refers to higher repayment efficacy (less default), – to less repayment efficacy (more default), ns to a non-significant result, and ∩ to an inverted-U shaped relationship. [d]p-values are reported exactly where provided in the original study, otherwise the level of significance they do or do not pass is reported. [e]Number of loan groups does not necessarily equal sample size of the analysis (e.g., if the analysis is based on individuals). [f]Numbers refer to mean, mean (standard deviation), mean (range) or range of lending group sizes in the sample, if not indicated otherwise. [g]Indicates the empirical approach reported by the authors (if any) that attempts to eliminate potential confounds between the predictor variable and repayment outcome in order to increase confidence in a causal effect of the predictor rather than a correlation. [h]We have excluded one study from this category as it tests the effect of kinship *or* coming from the same province combined into a single predictor variable and the influence of kinship and geographical proximity cannot be separated. The study reports a positive effect of this variable on repayment (Chen et al., 2020). [*]The authors report a positive association when the analysis is based on the response of the group leader.

SM2: Summary of review results for all categories of predictors

Table S2. Number, direction and proportion of the associations of proxies for evolutionary mechanisms of cooperation (i.e. category of predictor variable) with the repayment outcome of group-based microfinance loans. Table is based on the 13 categories into which predictor variables were grouped (see main text and Table S1 above). The rows in bold font aggregate the percentages and proportions of all categories grouped into a particular mechanism for the evolution of cooperation (note that "common ancestry" only has a single category). Numbers in table represent percentage of associations and numbers in parentheses represent proportion of associations. Positive associations are in line with our evolutionary hypotheses, negative associations are not in line, non-significant are statistically non-significant, and inverted-U refer to an inverted-U shaped relationship. Note that some columns do not add up exactly to 100% due to rounding.

**Category of predictor variable**	**Direction of association with repayment outcome**			
	**Negative**	**Non-significant**	**Inverted U**	**Positive**
Family members in group	50% (4/8)	38% (3/8)	0% (0/8)	12% (1/8)
**Common ancestry**	**50% (4/8)**	**38% (3/8)**	**0% (0/8)**	**12% (1/8)**
Prior acquaintance of group members	10% (1/10)	40% (4/10)	0% (0/10)	50% (5/10)
Personal and business relations between group members	21% (4/19)	53% (10/19)	0% (0/19)	26% (5/19)
Tenure and continuity of group composition	37% (7/19)	47% (9/19)	0% (0/19)	16% (3/19)
Frequency of group meetings	20% (1/5)	20% (1/5)	0% (0/5)	60% (3/5)
Geographic proximity of group members	0% (0/17)	53% (9/17)	6% (1/17)	41% (7/17)
Participation in community and other associations	22% (2/9)	33% (3/9)	0% (0/9)	44% (4/9)
Shared extra-group social ties	0% (0/1)	0% (0/1)	0% (0/1)	100% (1/1)
**Prior interaction**	**19% (15/80)**	**45% (36/80)**	**1% (1/80)**	**35% (28/80)**
Self-formation of group	0% (0/6)	33% (2/6)	0% (0/6)	67% (4/6)
Rejection and invitation of members	20% (2/10)	50% (5/10)	0% (0/10)	30% (3/10)
**Partner choice**	**13 % (2/16)**	**44% (7/16)**	**0% (0/16)**	**44% (7/16)**
Socio-cultural homogeneity of group	17% (1/6)	17% (1/6)	0% (0/6)	67% (4/6)
Socio-economic homogeneity of group	23% (6/26)	58% (15/26)	4% (1/26)	15% (4/26)
Rurality and limited mobility	33% (2/6)	17% (1/6)	0% (0/6)	50% (3/6)

Supplementary references

Ahlin, C., and Townsend, R. M. (2007). Using Repayment Data to Test Across Models of Joint Liability Lending. *The Economic Journal*, *117*(517), F11–F51. https://doi.org/10.1111/j.1468-0297.2007.02014.x

Al-Azzam, M., Carter Hill, R., and Sarangi, S. (2012). Repayment performance in group lending: Evidence from Jordan. *Journal of Development Economics*, *97*(2), 404–414. https://doi.org/10.1016/j.jdeveco.2011.06.006

Amwayi, E. A., Omete, F. I., and Asakania, F. M. (2014). Analysis of Group Based Loan Default in Kenya: The Case of Agricultural Finance Corporation of Kenya. *IOSR Journal of Economics and Finance*.

Anthony, D. (2005). Cooperation in Microcredit Borrowing Groups: Identity, Sanctions, and Reciprocity in the Production of Collective Goods. *American Sociological Review*, *70*(3), 496–515. https://doi.org/10.1177/000312240507000307

Anthony, D., and Horne, C. (2003). Gender and cooperation: Explaining loan repayment in micro-credit groups. *Social Psychology Quarterly*, *66*(3), 293–302.

Asgedom, A. K. (2015). Determinants of MFIs Group Loan Repayment Performance: A Case of Dedebit Credit and Saving Institution, Mekelle, Ethiopia. *Journal of Poverty, Investment and Development*, *10*(0), 1-21–21.

Ayogyam, A., Mohammed, H., Goddana, M. D., and Boateng, E. (2014). Monitoring loan repayment among farmers in Techiman, Ghana: Investigating the effect of cooperative farming system. *Journal of Emerging Trends in Economics and Management Sciences*, *5*(1), 32–37.

Baland, J.-M., Somanathan, R., and Vandewalle, L. (2008). Microfinance Lifespans: A Study of Attrition and Exclusion in Self-Help Groups in India. *India Policy Forum*, *4*(1), 159–210.

Bassem, B. (2008). Determinants of successful group loan repayment: An application to Tunisia. *Journal of Sustainable Development in Africa*, *10*(2), 766–800.

Bhatt, N., and Tang, S.-Y. (2002). Determinants of Repayment in Microcredit: Evidence from Programs in the United States. *International Journal of Urban and Regional Research*, *26*(2), 360–376. https://doi.org/10.1111/1468-2427.00384

Bumbie, M. Y. (2013). *Determinants of Repayment Performance of Group and Individual Lending in Microfinance: A Case Study in the Upper West Region of Ghana*. University of Ghana.

Carpenter, J., and Williams, T. (2014). Peer Monitoring and Microcredit: Field Experimental Evidence from Paraguay. *Oxford Development Studies*, *42*(1), 111–135. https://doi.org/10.1080/13600818.2014.887061

Chen, Y., Deng, K., and Xu, Z. (2020). The social capital in microfinance issued by commercial banks: Evidence from China. *Applied Economics Letters*, *27*(4), 349–352. https://doi.org/10.1080/13504851.2019.1616054

Dalla Pellegrina, L., De Michele, A., Di Maio, G., Landoni, P., and Parravicini, S. (2017). Group Meeting Frequency and Borrowers' Repayment Performance in Microfinance: Evidence from a Quasi-Natural Experiment in South Africa. *University of Milan Bicocca Department of Economics, Management and Statistics Working Paper No. 374*. http://dx.doi.org/10.2139/ssrn.3080201

Giné, X., and Karlan, D. (2014). Group versus individual liability: Short and long term evidence from Philippine microcredit lending groups. *Journal of Development Economics*, *107*, 65–83. https://doi.org/10.1016/j.jdeveco.2013.11.003

Godquin, M. (2004). Microfinance Repayment Performance in Bangladesh: How to Improve the Allocation of Loans by MFIs. *World Development*, *32*(11), 1909–1926. https://doi.org/10.1016/j.worlddev.2004.05.011

Griffin, D., and Husted, B. W. (2015). Social sanctions or social relations? Microfinance in Mexico. *Journal of Business Research*, *68*(12), 2579–2587. https://doi.org/10.1016/j.jbusres.2015.06.023

Guttman, J. M. (2007). Repayment Performance in Microcredit Programs: Theory and Evidence. *Networks Financial Institute Working Paper*, *2007-WP-11*. https://www.indstate.edu/business/sites/business.indstate.edu/files/Docs/2007-WP-11_Guttman.pdf

Hermes, N., Lensink, R., and Mehrteab, H. T. (2005). Peer Monitoring, Social Ties and Moral Hazard in Group Lending Programs: Evidence from Eritrea. *World Development*, *33*(1), 149–169. https://doi.org/10.1016/j.worlddev.2004.09.001

Kangogo, D., Lagat, J., and Ithinji, G. (2013). *The Influence of Social Capital Dimensions on Household Participation in Micro-Credit Groups and Loan Repayment Performance in Uasin Gishu County, Kenya*. Egerton University.

Karlan, D. (2007). Social connections and group banking. *The Economic Journal*, *117*(517), F52–F84. https://doi.org/10.1111/j.1468-0297.2007.02015.x

Kasarjyan, M., Fritzsch, J., Buchenrieder, G., and Korff, R. (2007). *Repayment performance under joint liability borrowing. Does social capital matter? Evidence from Armenia*. 104th EAAE-IAAE Seminar Agricultural Economics and Transition.

Kinyondo, A. A., and Okurut, N. (2009). Determinants of loan repayment performance in microcredit institutions: Evidence from Tanzania. *Asia-African Journal of Economics and Econometrics*, *9*(2).

Kolstad, I., Pires, A. J. G., and Wiig, A. (2017). Within-group heterogeneity and group dynamics: Analyzing exit of microcredit groups in Angola. *Oxford Development Studies*, *45*(3), 338–351. https://doi.org/10.1080/13600818.2016.1243237

Kritikos, A. S., and Vigenina, D. (2005). Key Factors of Joint-Liability Loan Contracts: An Empirical Analysis. *Kyklos*, *58*(2), 213–238. https://doi.org/10.1111/j.0023-5962.2005.00286.x

Mahmud, M. (2019). Repaying Microcredit Loans: A Natural Experiment on Liability Structure. *The Journal of Development Studies*, *0*(0), 1–16. https://doi.org/10.1080/00220388.2019.1632432

Mason, D. R. (2011). *Cooperation as Collateral? Social Capital and Joint Liability Microfinance Group Lending in Nicaragua*. University of California, Los Angeles.

Matta, D. (2004). *Examining Determinants of Group Loan Repayment in the Dominican Republic* [Ohio University]. http://rave.ohiolink.edu/etdc/view?acc_num=ohiou1090935410

Noglo, Y., and Androuais, A. (2015). The determinants of group lending repayment performance: Evidence from Togo. *Canadian Journal of Development Studies / Revue Canadienne d'études Du Développement*, *36*(4), 536–554. https://doi.org/10.1080/02255189.2015.1046372

Oke, J. T. O., Adeyemo, R., and Agbonlahor, M. U. (2007). An Empirical Analysis of Microcredit Repayment in Southwestern Nigeria. *Journal of Human Behavior in the Social Environment*, *16*(4), 37–55. https://doi.org/10.1300/10911350802081592

Olomola, A. S. (2002). Social capital, microfinance group performance and poverty implications in Nigeria. *Ibadan, Nigeria: Nigerian Institute of Social and Economic Research*, 1–25.

Paxton, J., Graham, D., and Thraen, C. (2000). Modeling Group Loan Repayment Behavior: New Insights from Burkina Faso. *Economic Development and Cultural Change*, *48*(3), 639–655. https://doi.org/10.1086/452613

Postelnicu, L., Hermes, N., and Servin, R. (2019). External Social Ties and Loan Repayment of Group Lending Members: A Case Study of Pro Mujer Mexico. *The Journal of Development Studies*, *55*(8), 1784–1798. https://doi.org/10.1080/00220388.2018.1464148

Sabin, N., and Reed-Tsochas, F. (2018). *Able but Unwilling to Enforce: Cooperative Dilemmas in Group Lending* (SSRN Scholarly Paper ID 3275526). Social Science Research Network. https://papers.ssrn.com/abstract=3275526

Sharma, M., and Zeller, M. (1997). Repayment performance in group-based credit programs in Bangladesh: An empirical analysis. *World Development*, *25*(10), 1731–1742. https://doi.org/10.1016/S0305-750X(97)00063-6

Simtowe, F., Zeller, M., and Phiri, A. (2006). Determinants of moral hazard in microfinance: Empirical evidence from joint liability lending programs in Malawi. *African Review of Money Finance and Banking*, 5–38.

Sungwacha, S. M., Singoro, B. W., and Kirathi, M. (2014). Factors influencing loan repayment performance among group borrowers in Bungoma West District, Bungoma County. *IOSR Journal of Business and Management*, *16*(7), 24–28.

van Bastelaer, T., and Leathers, H. (2006). Trust in Lending: Social Capital and Joint Liability Seed Loans in Southern Zambia. *World Development*, *34*(10), 1788–1807.

Wenner, M. D. (1995). Group credit: A means to improve information transfer and loan repayment performance. *The Journal of Development Studies*, *32*(2), 263–281. https://doi.org/10.1080/00220389508422414

Wydick, B. (1999). Can Social Cohesion be Harnessed to Repair Market Failures? Evidence from Group Lending in Guatemala. *The Economic Journal*, *109*(457), 463–475. https://doi.org/10.1111/1468-0297.00457

Zeller, M. (1998). Determinants of Repayment Performance in Credit Groups: The Role of Program Design, Intragroup Risk Pooling, and Social Cohesion. *Economic Development and Cultural Change*, *46*, 599–620. https://doi.org/10.1086/452360
